# Fibroblast Growth Factor 8 Suppresses Neurotoxic Astrocytes and Alleviates Neuropathic Pain via Spinal FGFR3 Signaling

**DOI:** 10.1007/s12264-025-01533-x

**Published:** 2025-10-30

**Authors:** Huizhu Liu, Lanxing Yi, Guiling Li, Kangli Wang, Hongsheng Wang, Yuqiu Zhang, Benlong Liu

**Affiliations:** 1 State Key Laboratory of Medical Neurobiology and MOE Frontiers Center for Brain Science, Institutes of Brain Science, Fudan University, Shanghai, 200032 China; 2 Department of Traditional Chinese Medicine, Songjiang Research Institute, Shanghai Key Laboratory of Emotions and Affective Disorders, Songjiang Hospital Affiliated to Shanghai Jiao Tong University School of Medicine, Shanghai, 201600 China

**Keywords:** Reactive astrocytes, FGF8, FGFR3 signaling, Microglia–astrocyte interaction, Neuropathic pain, Spinal dorsal horn

## Abstract

Astrocytes in the spinal dorsal horn (SDH) exhibit diverse reactive phenotypes under neuropathic conditions, yet the mechanisms driving this diversity and its implications in chronic pain remain unclear. Here, we report that spared nerve injury (SNI) induces marked upregulation of both complement component 3 (C3⁺, A1-like) and S100 calcium-binding protein A10 (S100A10⁺, A2-like) astrocyte subpopulations in the SDH, with elevated microglial cytokines including interleukin-1α, tumor necrosis factor-α, and complement component 1q. Transcriptomic, immunohistochemical, and Western blot analyses reveal co-activation of multiple reactive astrocyte states over a unidirectional shift toward an A1-like phenotype. Fibroblast growth factor 8 (FGF8), a neuroprotective factor via FGFR3, mitigated microglia-induced C3⁺ astrocyte reactivity in vitro and suppressed spinal C3 expression and mechanical allodynia following intrathecal administration in SNI mice. These findings reveal a microglia–astrocyte signaling axis that promotes A1 reactivity and position FGF8 as a promising therapeutic candidate for neuropathic pain by modulating astrocyte heterogeneity.

**Supplementary Information:**

The online version contains supplementary material available at 10.1007/s12264-025-01533-x.

## Introduction

Astrocytes are the most abundant glial cells in the central nervous system (CNS), providing structural and metabolic support to neurons and playing essential roles in various neural processes [1, 2]. Traditionally considered homogeneous, accumulating evidence reveals that astrocytes exhibit substantial diversity in morphology, physiology, and function across brain regions [3–6]. In response to CNS damage or disease, astrocytes undergo reactive transformations, contributing to neurological disorders. Genetic profiling of reactive astrocytes in models of ischemic stroke and neuroinflammation has identified at least two distinct subtypes: neurotoxic A1 astrocytes, which lose supportive functions and gain synapse-damaging properties, and neuroprotective A2 astrocytes, associated with tissue repair [7, 8]. A1 astrocytes have been implicated in various CNS diseases such as depression [9–11], cognitive dysfunction [10], ischemic stroke [12], and neurodegenerative disorders [13–15]. In neuropathic pain, astrocytes in the SDH undergo significant morphological, molecular, and functional changes [1, 2, 16]. Recent studies reveal that reactive astrocytes in the SDH are heterogeneous and contribute differentially to chronic pain processing [17–20]. However, the mechanisms underlying this phenotypic diversity in astrocytes in neuropathic pain remain poorly understood.

Microglia-derived pro-inflammatory cytokines, including interleukin-1α (IL-1α), tumor necrosis factor-α (TNF-α), and complement component 1q (C1q), have been shown to play a pivotal role in inducing the neurotoxic reactive astrocyte [7], yet effective molecular strategies to counteract this transformation are still lacking. FGF8, a member of the FGF family, plays critical roles in cellular differentiation, neuronal survival, and tissue development [21]. FGF8 primarily exerts its biological functions through binding to FGF receptors 2 and 3 (FGFR2 and FGFR3), among which FGFR3 is highly enriched in spinal astrocytes [22]. Notably, FGF8 exhibits a high affinity for FGFR3 [23–25] and is critical for maintaining cortical astrocytes in a nonreactive state [26, 27]. In this study, we investigate the role of spinal FGF8-FGFR3 signaling in modulating neurotoxic astrocytes in a SNI model of neuropathic pain. We hypothesize that FGF8 may attenuate A1 astrocyte phenotype and thereby alleviate neuropathic pain.

## Materials and Methods

### Animals

Adult male C57BL/6 mice (8–10 weeks) were purchased from the Shanghai Experimental Animal Center of the Chinese Academy of Sciences. All animals were housed under a 12 h light/dark cycle at 22°C, ~60% humidity, and ~100 lux illumination, with food and water provided *ad libitum*. Animals were randomly assigned to experimental and control groups, and all behavioral testing was performed by an investigator blinded to group assignments and treatments. All animal procedures were approved by the Institutional Animal Care and Use Committee of Fudan University (Permit Number: SYXK2009-0082) and conducted following the guidelines of the International Association for the Study of Pain. Mice were euthanized by carbon dioxide inhalation upon completion of experiments.

### Rodent Models of Acute and Chronic Pain

We employed three established mouse models to investigate acute and chronic pain states. (1) SNI was used to induce long-lasting neuropathic pain, following previously described procedures [28]. Briefly, mice were anesthetized with pentobarbital sodium (50 mg/kg i.p.), and the sciatic nerve branches—the common peroneal, tibial, and sural nerves—were exposed. The common peroneal and tibial nerves were tightly ligated with 6-0 silk sutures and transected distal to the ligation site, leaving the sural nerve intact. In the sham group, the sciatic nerve and its terminal branches were exposed without ligation or transection. (2) Capsaicin-induced acute pain was evoked by intraplantar injection of capsaicin (5 μg in 10 μL, MCE HY-10448R) [29]. Spontaneous nocifensive behaviors (paw licking, flinching, lifting) were recorded over a 5-min observation period. (3) Complete Freund’s adjuvant (CFA, 20 μL, Sigma F5881) was injected into the plantar surface of the hind paw to induce persistent inflammatory pain [30]. Behavioral assessments were conducted 24 h post-injection.

### *von* Frey Test

Animals were habituated to the testing environment for at least 2 days before baseline testing, with room temperature and humidity maintained consistently throughout the procedure. The *von* Frey test for mechanical sensitivity was conducted by measuring paw withdrawal thresholds (PWTs) in response to a series of calibrated *von* Frey hairs (0.04–2 g, Stoelting Company). Mice were placed in a chamber with an elevated metal mesh floor for 30 min before testing. The *von* Frey filaments were applied to the central region of the hindpaw plantar surface in ascending order, with each filament applied five times for 2 s, interspersed by 15-s intervals. A positive response was defined as a withdrawal from at least three out of five applications, and the PWT was recorded in grams. For response frequency determination, filaments (0.04 g, 0.07 g, 0.16 g) were applied 10 times each at 10-s intervals in ascending order of force to assess the response frequency.

### Hargreaves Test

Thermal nociceptive sensitivity was assessed using the Hargreaves test (IITC Life Science Instruments). Mice were individually placed into Plexiglas chambers on an elevated glass platform and allowed to acclimate for 30 min. A radiant heat source was then applied to the plantar surface of the hind paw, and paw withdrawal latency (PWL) was recorded. The stimulus intensity was calibrated to yield a baseline PWL of 10–14 s. To prevent tissue damage, a cutoff time of 20 s was imposed.

### Immunofluorescence Staining and Microglia Morphological Analysis

Mice were euthanized with an overdose of urethane and transcardially perfused with warm normal saline, followed by ice-cold 4% paraformaldehyde in 0.1 mol/L phosphate buffer (pH 7.4), post-fixed in the same fixative for 2 h at 4 °C, and cryoprotected overnight in 30% sucrose PBS-buffered solution at 4 °C. Spinal cord tissue was sectioned into 35 μm slices using a freezing microtome (Leica Microsystems). Free-floating sections were incubated in blocking solution containing 10% donkey serum and 0.3% Triton X-100 for 2 h at room temperature (RT), followed by overnight incubation at 4 °C with primary antibodies (see Supporting Information Table S1 for details). After washing with PBS (three times for 10 min), sections were incubated with secondary antibodies for 2 h at RT. After washing with PBS (three times for 10 min), sections were mounted onto slides. For double-labeling, combinations of primary and secondary antibodies were applied simultaneously. Specificity of immunostaining was confirmed by omission of primary antibodies or pre-absorption with the corresponding blocking peptide. Immunofluorescence images were captured using a confocal laser-scanning microscope (Olympus FV3000, Tokyo, Japan). The fluorescence intensity of IBA-1, GFAP, C3, S100A10, VGLUT2, and PSD95 was quantified using ImageJ software (NIH, Bethesda, MD, USA). For each animal (*n =* 3 per group), at least three randomly selected sections were analyzed. The number of double-labeled cells (C3^+^GFAP^+^, S100A10^+^GFAP^+^, FGFR3^+^GFAP^+^, and PLCE^+^GFAP^+^) and total GFAP^+^ astrocytes in the superficial dorsal horn (laminae I-III) under a 20× objective were quantified using ImageJ software (NIH, Bethesda, MD, USA).

Microglial morphological analysis was conducted using Z-stack images acquired from IBA-1-immunopositive microglia acquired with a confocal microscope (Olympus FV3000) under a 40× objective at 3-μm step intervals. Consecutive Z-stack images were reconstructed into maximum intensity projection images using Fiji software. Sholl analysis was performed by drawing concentric circles centered on the microglial soma, starting at a 5-μm radius and increasing in 5-μm increments. The number of intersections between microglial processes and concentric circles was counted to generate a Sholl plot. Additional parameters, including process branch length and soma area, were also quantified for each cell.

### Western Blotting

Protein samples were prepared from L2–L5 spinal cord segments and homogenized in lysis buffer (Beyotime, P0013B) supplemented with protease inhibitors (Roche Diagnostics, 05892791001) and phosphatase inhibitors (Roche Diagnostics, 04906837001). Equal amounts of protein were loaded onto 10% SDS-PAGE gels (Beyotime, P0012A) for electrophoretic separation and transferred to PVDF membranes (Millipore, IPVH00010). Membranes were blocked with 5% nonfat milk in Tris-buffered saline (pH 7.6) with 0.1% Tween-20 for 2 h at RT and subsequently incubated overnight at 4°C with primary antibodies. Afterward, membranes were incubated with HRP-conjugated secondary antibodies for 2 h at room temperature. GAPDH was used as a loading control. Protein bands were visualized using enhanced chemiluminescence (Thermo Fisher Scientific, 34095) and imaged with a ChemiDoc XRS system (Bio-Rad). Antibody sources are detailed in Supporting Information Table S1. Each experiment was repeated three to four times for reproducibility, and band intensity was quantified using Image Lab software (Bio-Rad).

### Intra-dorsal Horn Injection and Adeno-Associated Virus

Intraspinal injections were performed following established protocols [31]. Mice were anesthetized with isoflurane (induction: 3%, maintenance: 1.0%–1.5%) and placed in a stereotaxic apparatus. The depth of anesthesia was carefully monitored throughout the procedure, ensuring loss of corneal reflex and absence of response to noxious paw pinch. Body temperature was maintained at 37–38 °C using a thermostatically regulated heating pad. The animal’s spine was fixed in a stereotaxic apparatus. After removal of paraspinal muscles on the right side, between the T13 and L1 vertebrae, a partial laminectomy was performed to expose the spinal cord. A small window was created to allow insertion of a glass micropipette (tip diameter: 10–30 µm) into the SDH. The pipette was tilted at a 45° angle and inserted approximately 300 μm lateral to the midline and 420 μm deep from the dura (corresponding to a vertical depth of 300 µm from the dura) to target the superficial dorsal horn. Adeno-associated viruses (AAVs) were injected at two sites within the right dorsal horn (600 nL per site) using an air pressure system (NANOLITER 2010 injecter, WPI, USA) at a rate of 40 nL/min. The pipette was left in place for an additional 10 min following each injection to prevent backflow. Short-hairpin RNA targeting FGFR3 (FGFR3 shRNA) or a scrambled control sequence (Ctrl shRNA) was designed and packaged by the Sunbio Medical Biotechnology Company (Shanghai, China). The AAV-FGFR3 shRNA construct was generated using an shRNA sequence reported in a prior study [32].

### Drug Administration

Recombinant FGF8 protein (0.5 μg or 1 μg in 5 μL PBS; R&D Systems, 423-F8-025/CF) was administered via intrathecal (i.t.) lumbar puncture between L5–L6 vertebrae under isoflurane anesthesia using a 31G needle-equipped Hamilton syringe (25 μL volume). A tail-flick response was used to confirm successful delivery. To assess the effect of FGFR2 inhibition or microglial activation [33]‌, Alofanib (10 μg in 5 μL PBS; Selleck, S8754) or lipopolysaccharide (LPS, 10 μg in 5 μL PBS; Sigma, L2880) was administered i.t. 1 h prior to FGF8 injection. Behavioral assessments were performed 1 h after FGF8 injection by experimenters blinded to group assignment. For proliferative labeling, 5-bromo-2'-deoxyuridine (BrdU; 100 mg/kg in PBS; Sigma, B5002) was injected intraperitoneally.

### Astrocyte Isolation and Cell Culture

Isolated cultures of the spinal cord were prepared from 1–2-day-old C57BL/6 mice. The tissue was minced and enzymatically dissociated using 0.125% trypsin at 37°C for 15 min, followed by gentle mechanical dissociation to obtain a single-cell suspension. The cell suspension was filtered through a 70-µm mesh, centrifuged at 1000 r/min for 5 min, and resuspended in DMEM/F12 (Gibco, 11330-032) supplemented with 10% fetal bovine serum (FBS, Gibco, 26140-079) and 1% penicillin/streptomycin (Gibco, 15140-122). Loosely attached microglia and oligodendrocyte precursor cells were removed by shaking the culture flasks on a rotary shaker at 260 r/min for 4–6 h at 37°C. The enriched astrocytes were then detached using trypsin-EDTA and subjected to various treatments. The purity of the astrocyte culture was assessed by immunostaining for GFAP.

To induce neurotoxic A1 astrocyte phenotype, purified astrocytes were cultured for 10 days and then treated for 24 h with IL-1α (3 ng/mL, Sigma, I3901), TNF-α (30 ng/mL, Cell Signaling Technology, 8902SF), and C1q (400 ng/mL, MyBioSource, MBS143105). A1 phenotype induction was confirmed by quantitative real-time PCR detection of *C3* and *S100a10*. As a positive control, astrocytes were activated with 100 ng/mL LPS (Sigma, L2880) or microglia-conditioned media (MCM), obtained from microglia treated with 100 ng/mL LPS for 24 h. To examine potential interventions in astrocyte transformation, FGF8 (100 ng/mL, R&D Systems, CAT # 423-F8-025/CF) was pre-incubated in the culture medium for 24 h. After treatment, the expression of *C3*, *S100a10*, and other genes was assessed by quantitative real-time PCR.

### Quantitative Real-Time PCR

Total RNA was isolated from L2–L5 spinal cord segments or cultured astrocytes using TRIzol reagent (Invitrogen, 15596-026) according to the manufacturer’s protocol. RNA was purified with chloroform extraction, precipitated with isopropanol, washed with 75% ethanol, and then eluted in ribonuclease-free water. Reverse transcription to complementary DNA (cDNA) was performed using the PrimeScript™ RT Reagent Kit (TAKARA, RR047A). Quantitative real-time polymerase chain reaction (qRT-PCR) was conducted using the TB Green™ Premix Ex Taq™ II kit (TAKARA, RR820A) on a QuantStudio 3 Real-Time PCR System (QuantStudio 3, Thermo). Each sample was analyzed in triplicate, and data were processed using QuantStudio software (Applied Biosystems). β-actin was used as an internal reference. Primer sequences are provided in Table S1. Relative gene expression was calculated from Ct values of the target and β-actin using the formula: relative expression 2^^−(Ct target − Ct β-actin)^.

### Isolation of Astrocytes and RNA Sequencing

Astrocytes from L2–L5 spinal cord segments were isolated using the Adult Brain Dissociation Kit (Miltenyi Biotec, 130-107-677). Briefly, tissues were enzymatically dissociated into single cells and resuspended in PBS containing 10% FBS. FcR Blocking Reagent was added, followed by incubation with Anti-ACSA-2 magnetic beads (Miltenyi Biotec, 130-097-678) at 4°C for 15 min in the dark. Cells were then centrifuged, and the pellet was resuspended in PBS containing 10% FBS. Next, the cell suspension was applied to an LS column, where astrocytes bound to the magnetic beads remained, while unbound cells were washed away. The sorted astrocytes were collected after a final PBS wash. The sorted astrocytes were collected after a final wash with PBS containing 10% FBS, with the supernatant removed.

Total RNA was extracted from isolated astrocytes using the TRIzol reagent. A cDNA library was constructed following the Illumina protocol and sequenced using an Illumina NovaSeq 6000 platform (read length 2×150 bp). Raw sequencing data were processed with Fastp (https://github.com/OpenGene/fastp) for quality control and adapter trimming. HISAT2 (http://ccb.jhu.edu/software/hisat2/index.shtml) and StringTie (https://ccb.jhu.edu/software/stringtie/) were used to evaluate the quality of transcriptome data, including sequencing saturation, gene coverage, distribution of Reads across the reference genome, and distribution analysis of Reads in different chromosomes. Gene expression levels were quantified using RSEM (http://deweylab.github.io/RSEM/) with TPM/FPKM as the units for consistency across samples. Differential gene expression analysis was performed using DESeq2 (http://bioconductor.org/packages/stats/bioc/DESeq2/), with a *P* value < 0.05 and |log2FC| >0.58 as thresholds for identifying differentially expressed genes (DEGs).

### Acute Spinal Cord Slice Preparation and Electrophysiological Recording

On day 14 post-SNI, lumbar spinal cords were rapidly dissected and immersed in ice-cold, oxygenated cutting artificial cerebrospinal fluid (ACSF). After removal of surrounding tissues and isolation of the lumbar enlargement, the spinal cord was embedded in agarose and mounted on a vibratome. Transverse slices (280 μm thick) were sectioned in ice-cold cutting ACSF continuously bubbled with 95% O₂ and 5% CO₂ (cutting parameters: amplitude 1 mm, speed 0.10 mm/s). Slices were incubated in oxygenated ACSF at 32°C for 30 min and then equilibrated at room temperature for at least 1 h before recording. During electrophysiological recordings, slices were perfused with standard ACSF (control group) or ACSF containing FGF8 (500 ng/mL; experimental group). For action potential (AP) recordings, neurons were held in current-clamp mode and stimulated with step currents ranging from − 50 pA to +150 pA in 10 pA increments (1 s duration per step). Data were analyzed using Clampfit 10.6. For spontaneous excitatory and inhibitory postsynaptic current (sEPSC and sIPSC) recordings, neurons were voltage-clamped at − 70 mV and 0 mV, respectively. Signals were recorded in gap-free mode for 5 min, and 3 min of stable traces were selected for analysis. Event frequency, amplitude, and cumulative probability distributions were analyzed using MiniAnalysis software.

### Statistical Analysis

Data are presented as mean ± standard error of the mean (SEM). All analyses were performed in a blinded manner, with no data excluded due to outliers, using GraphPad Prism 8.0 (GraphPad Software, San Diego, CA, USA). Sample sizes for behavioral experiments were determined based on previous experience and power calculations (power: 0.75; alpha: 0.05) using G Power, with a minimum of four animals per group (*n* ≥ 4) required to detect significant effects. Normality and homogeneity of variance were verified using the Shapiro-Wilk and Levene tests (no data transformation was applied). Behavioral, immunohistochemistry, and Western blot data were analyzed using Student’s *t*-test or the Mann-Whitney U test (for nonparametric data) for two-group comparisons. For multiple-group comparisons, one-way or two-way repeated-measures (RM) ANOVA, or Kruskal-Wallis H tests (for nonparametric data) were performed, followed by *post hoc* Holm-Sidak or Student-Newman-Keuls tests. All statistical analyses were two-tailed, and a *P*-value of <0.05 was considered statistically significant. Sample sizes and statistical details are provided in the figure legends.

## Results

### Reactive Astrocytes Exhibit Distinct Phenotypic Changes in the SDH Following SNI

**Fig. 1:**
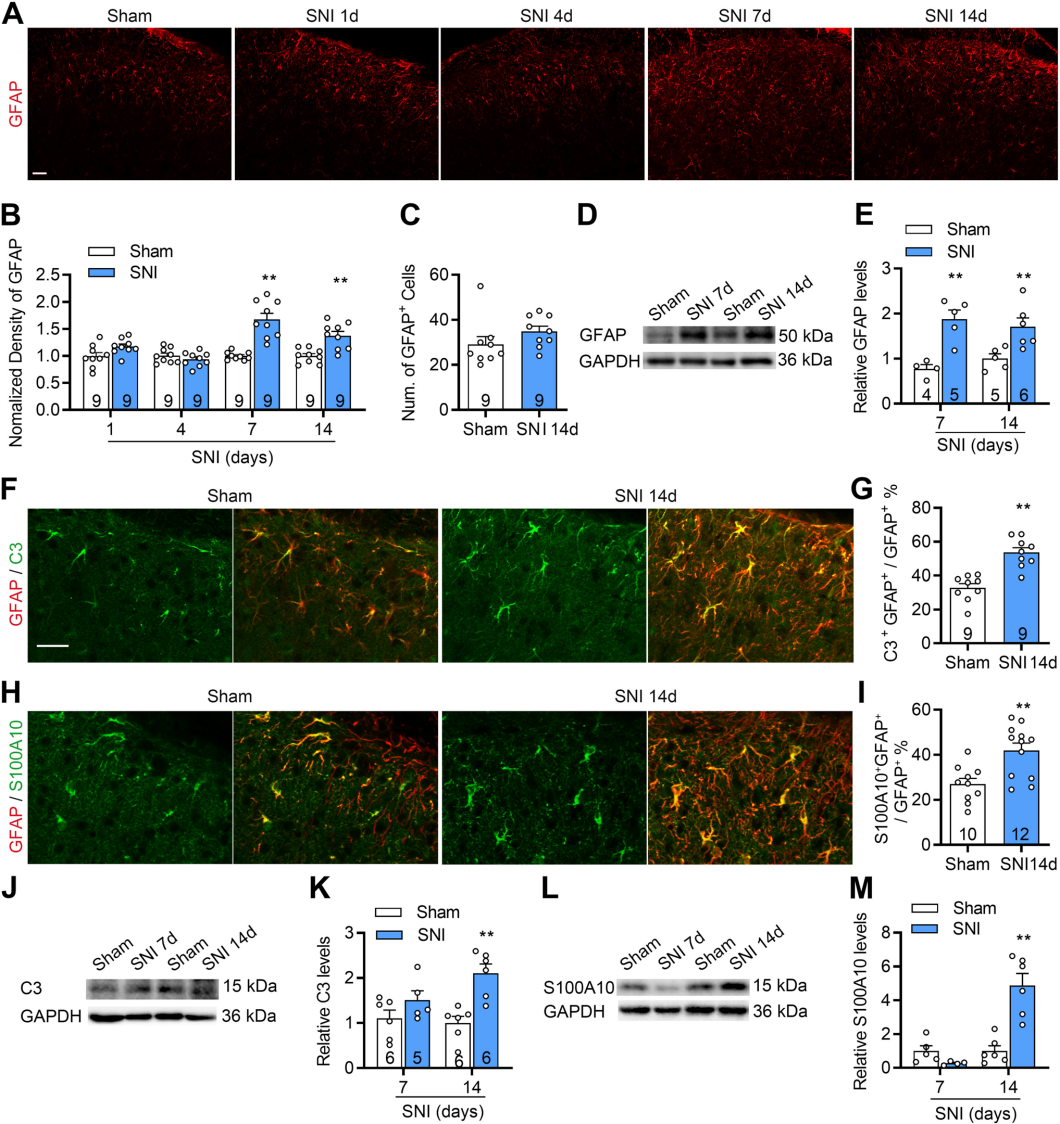
SNI induces activation of both neurotoxic and neuroprotective astrocyte phenotypes in the SDH. **A**, **B** Immunofluorescence staining of GFAP (an astrocytic marker) shows increased GFAP-immunoreactivity (GFAP-IR) in the ipsilateral spinal dorsal horn at days 7 and 14 after SNI. **C** Quantification showing unchanged numbers of GFAP^+^ astrocytes. **D**, **E** Representative Western blot images and quantification showing increased GFAP protein expression. **F**, **G** Representative images showing co-localization of C3 with GFAP in the SDH of sham and SNI mice (**F**) and quantification (**G**) on day 14 post-SNI. **H**, **J** Representative images showing co-localization of S100A10 with GFAP in the SDH of sham and SNI mice (**H**) and quantification (**I**) on day 14 post-SNI. **J**–**M** Representative Western blot images and quantification of C3 (**J**, **K**) and S100A10 (**L**, **M**) in the L2–L5 spinal cord segments on day 14 after SNI. *n* = 9–12 slices (**B**, **C**, **G**, **I**) from 3 mice; *n* = 4–6 mice (**E**, **K**, **M**). Scale bar, 25 μm (**A**, **F**). Statistics by two-way ANOVA followed by *post hoc* Holm-Sidak test (**B**, **E**, **K**, **M**), Mann-Whitney U test (**C**), and two-tailed Student’s *t*-test (**G**, **I**). **P < 0.01. Data are presented as mean ± SEM unless otherwise specified. Sample sizes are indicated in the figure or described in the legend where applicable.

Recent studies have classified reactive astrocytes into neurotoxic (A1) and neuroprotective (A2) phenotypes [7, 8], both of which are implicated in neuropathic pain mechanisms. To investigate astrocytic activation in the SDH following SNI, we assessed the expression of glial fibrillary acidic protein (GFAP). Immunofluorescence analysis revealed a significant upregulation of GFAP expression in the ipsilateral SDH from days 7 to 14 post-SNI compared to sham controls (Fig. 1A, B). Consistently, Western blot analysis confirmed that GFAP protein levels were significantly elevated at both day 7 and day 14 (Fig. 1D, E). We next examined astrocytic phenotypic transitions by analyzing the expression of C3, a marker of neurotoxic A1 astrocytes, and S100A10, a marker of neuroprotective A2 astrocytes. On day 14 post-SNI, immunofluorescence staining showed a marked increase in the proportion of C3^+^GFAP^+^ and S100A10^+^GFAP^+^ astrocytes in the superficial SDH (Fig. 1F–I). Western blot analysis further revealed that C3 and S100A10 protein levels were significantly upregulated at day 14, whereas changes at day 7 were not significant (Fig. 1J–M). In contrast, the total number of GFAP^+^ astrocytes remained unchanged (Fig. 1C), indicating that the observed phenotypic changes were likely independent of astrocyte proliferation.

**Fig. 2:**
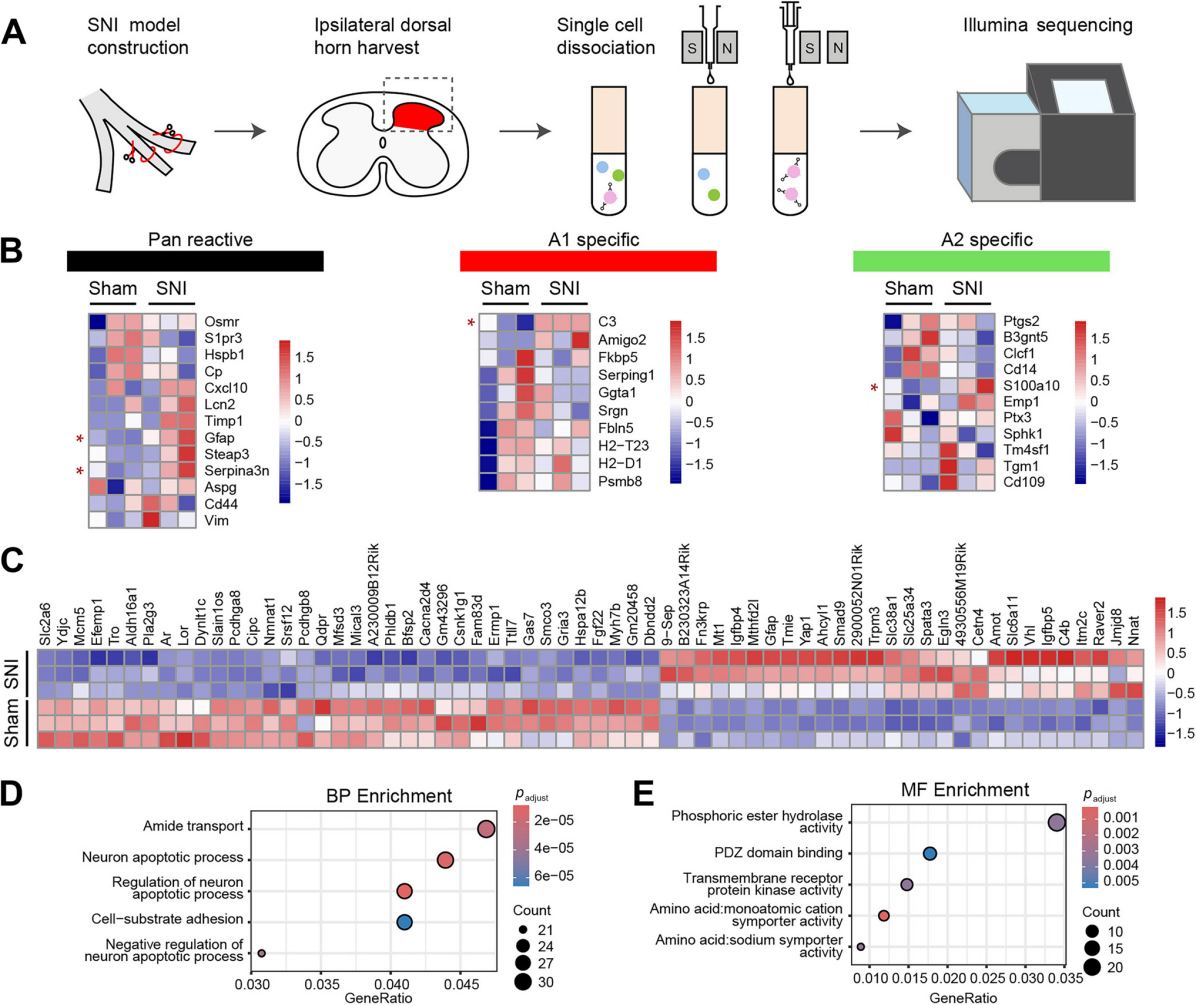
SNI induces transcriptomic signatures of reactive astrocytes in the spinal dorsal horn. **A** Schematic illustration of the experimental workflow corresponding to panels B–E. **B** Heatmaps showing expression changes of marker genes for pan-reactive, A1-specific, and A2-specific astrocyte phenotypes in sham and SNI samples. Genes marked with red asterisks were significantly up-regulated. **C** Hierarchical clustering of RNA-seq data reveals clear segregation between SNI and sham samples (36 upregulated, 28 downregulated genes; *P* < 0.05, |log₂FC| > 1.5). **D**, **E** Top 5 overrepresented Gene Ontology (GO) terms in the Biological Process (BP) (**D**) and Molecular Function (MF) (**E**) categories, based on differentially expressed genes identified in SNI samples compared to sham controls. *n* = 3 pooled samples per group (2 mice per sample).

Given the histological evidence for increased C3 expression and other reactive markers in spinal astrocytes post-SNI, we next investigated whether corresponding transcriptional changes underlie these phenotypic transitions. To this end, we performed RNA sequencing (RNA-seq) on astrocytes isolated from the SDH on day 14 post-SNI and from sham-operated controls (Fig. 2A, Fig. S1). Differential expression analysis (*P* value < 0.05 and |log2FC| >0.58) identified 402 upregulated and 371 downregulated genes in the SNI group (Fig. S1C). Transcriptomic profiling revealed a significant enrichment of pan-reactive, A1-specific, and A2-specific astrocyte signatures (Fig. 2B), consistent with the heterogeneity observed in immunohistochemical staining. To further characterize these alterations, Gene Ontology (GO) analysis of differentially expressed genes (DEGs, *P* value < 0.05 and |log2FC| >1.5) showed enrichment in biological processes such as amide transport, regulation of neuronal apoptosis, and cell-substrate adhesion (Fig. 2C, D). These processes are consistent with the known cytotoxic properties of A1 astrocytes, which have been implicated in promoting neuronal damage in neurodegenerative conditions [7, 8]. Additionally, molecular function analysis highlighted increased representation of genes involved in phosphoric ester hydrolase activity, PDZ domain binding, and transmembrane receptor protein kinase activity (Fig. 2E), further supporting a functional reprogramming of astrocytes in response to SNI.

### FGF8 Treatment Inhibits A1 Astrocyte Transition and Alleviates Mechanical Allodynia in the SNI Model

**Fig. 3:**
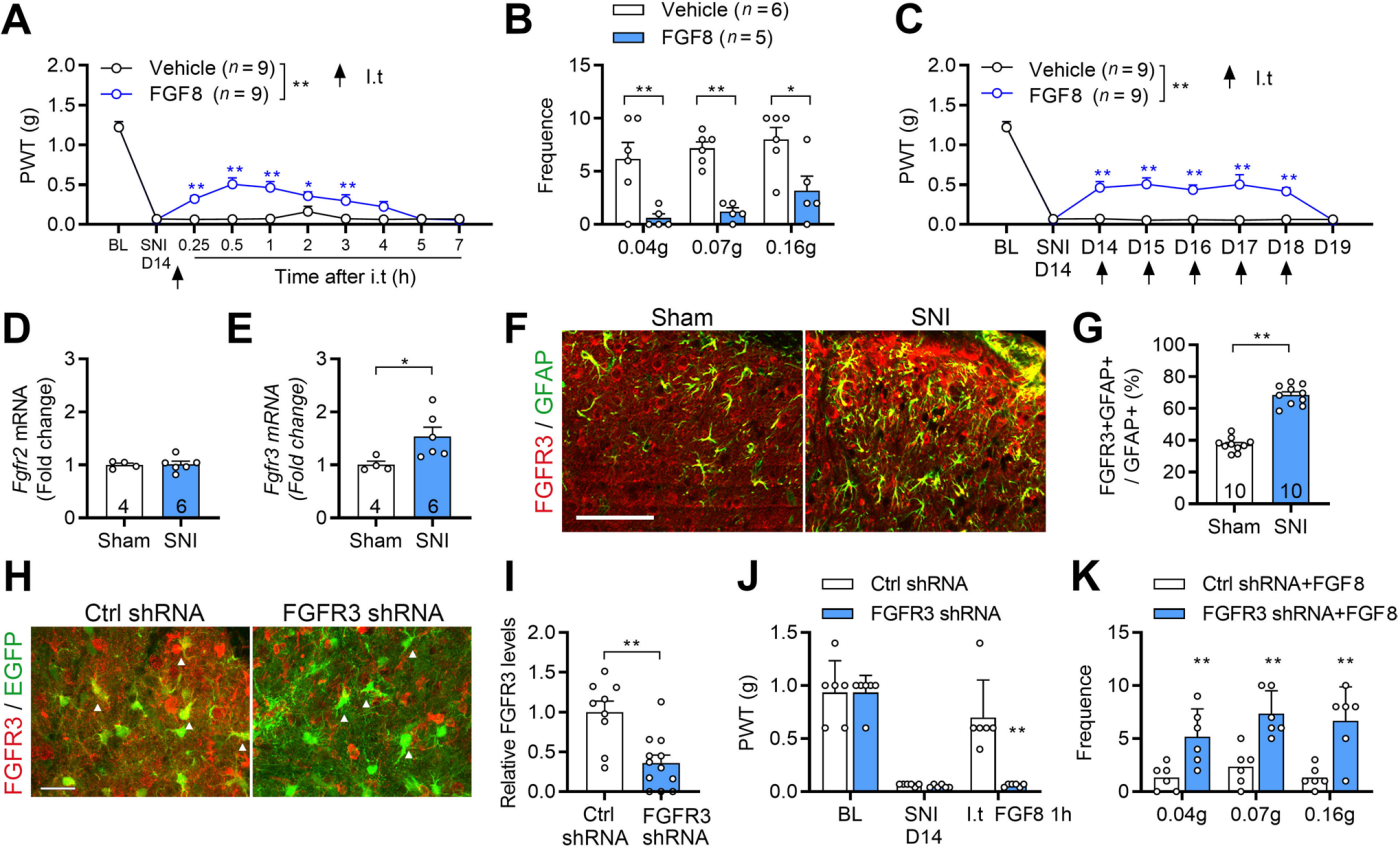
FGF8 alleviates SNI-induced mechanical allodynia through FGFR3. **A**, **B** Paw withdrawal threshold (**A**) and *von* Frey–evoked response frequency (**B**) in SNI mice following a single intrathecal injection of FGF8 (1 μg in 5 μL) on day 14 post-SNI. **C** Time course of mechanical allodynia response over five consecutive days following FGF8 administration on day 14 post-SNI. **D**, **E** qRT-PCR analysis showed changes of *Fgfr2* (**D**) and *Fgfr3* (**E**) in spinal cord tissue on day 14 post-SNI. **F**, **G** Representative images showing co-localization of FGFR3 with GFAP in the SDH of sham and SNI mice (**F**) and quantification (**G**) on day 14 post-SNI. **H**, **I** Representative images and quantification of FGFR3 knockdown efficiency in spinal astrocytes using FGFR3-targeting shRNA. Arrowheads indicate the typical cell. **J**, **K** Selective knockdown of FGFR3 attenuated the analgesic effects of intrathecal FGF8 injection on day 14 post-SNI. *n* = 9 mice (**A**, **C)**; *n* = 4–6 mice (**B–E**, **J**, **K**); *n* = 10-12 slices (**G**, **I**) from 3 mice. Scale bar: 25 μm (**F**, **H**). Statistics by two-way ANOVA followed by *post hoc* Holm-Sidak test (**A–C**, **J**, **K**) and two-tailed Student’s *t*-test (**D–I**). *P < 0.05, **P < 0.01. Data are presented as mean ± SEM. I.t, intrathecal injection.

To evaluate the therapeutic potential of FGF8 in neuropathic pain, we first examined its effects on mechanical allodynia in the SNI model. SNI induced a significant reduction in mechanical withdrawal thresholds from day 4 to day 14 compared to sham controls (Fig. S3A). A single intrathecal injection of FGF8 significantly alleviated mechanical allodynia in SNI mice, as evidenced by increased paw withdrawal thresholds (Fig. 3A, S3B) and reduced response frequency to *von* Frey filaments (Fig. 3B). The analgesic effect of 0.5 µg FGF8 diminished within 2 h, whereas the 1 µg dose elicited a more robust effect lasting for at least 3 h (Fig. S3B). Notably, FGF8 (1 µg) also significantly reduced nocifensive response in inflammatory pain models induced by CFA and capsaicin (Fig. S3D, E), suggesting broader analgesic potential beyond neuropathic pain. Based on these findings, the 1 µg dose was used in subsequent experiments. Repeated administration of FGF8 (1 µg/day) for three or five consecutive days beginning on day 14 post-SNI produced comparable analgesic effects (Fig. 3C, Fig. S3C). However, mechanical allodynia returned to pre-treatment levels 24 h after the final injection (Fig. 3C), indicating that continuous dosing is required to sustain analgesia. We next investigated whether receptor-level changes underlie the analgesic effects of FGF8. Quantitative PCR analysis revealed significant upregulation of *Fgfr3,* but not *Fgfr2,* in the SDH on day 14 post-SNI (Fig. 3D, E). To characterize the cellular distribution of *Fgfr3*, we analyzed single-nucleus RNA sequencing data from the adult mouse spinal cord [22], which revealed that *Fgfr2* is broadly expressed across multiple cell types, whereas *Fgfr3* expression is enriched in astrocytes (Fig. S2). Immunostaining further revealed an increased proportion of FGFR3^+^GFAP^+^ astrocytes in the SDH 14 days post-SNI (Fig. 3F, G). Notably, FGFR3 was also detectable in GFAP^−^ cells (Fig. 3F), indicating that FGFR3 is enriched in but not restricted to astrocytes. To determine whether the analgesic effects of FGF8 are mediated via astrocytic FGFR3, we selectively knocked down *Fgfr3* in spinal astrocytes using an AAV2/5-GFAabc1d-EGFP-FGFR3-shRNA virus (Fig. 3H, I). In mice with astrocyte-specific *Fgfr3* knockdown, FGF8 treatment failed to alleviate SNI-induced allodynia, demonstrating that astrocytic FGFR3 is essential for the analgesic effect (Fig. 3J, K). By contrast, pharmacological blockade of FGFR2 had no impact on FGF8-induced analgesia (Fig. S3F, G), indicating a minimal role for FGFR2 in mediating the analgesic effects of FGF8.

To further investigate downstream signaling activated by astrocytic FGF8-FGFR3 interaction, we examined the expression of phospholipase C epsilon 1 (PLCE1), a phospholipase regulated by Ras guanine nucleotide exchange factors [34]. FGF8 treatment significantly increased the proportion of PLCE1⁺GFAP⁺ astrocytes in the SDH 14 days post-SNI (Fig. S3H, I), suggesting that FGF8-FGFR3 signaling activates astrocytic PLCE1 under neuropathic conditions. Given the prominent role of FGF8-FGFR3 signaling in astrocyte modulation, we further examined its effect on astrocytic phenotypic transitions. Co-immunostaining showed that in sham animals, approximately 35.8% of GFAP⁺ astrocytes co-expressed C3 and FGFR3 (Fig. S4). In SNI mice, FGF8 treatment significantly reduced the proportion of C3⁺ astrocytes without affecting the proportion of S100A10⁺ astrocytes (Fig. S5A–F). These results indicate that FGF8 preferentially suppresses the transition to neurotoxic A1 astrocytes while sparing neuroprotective A2 phenotypes.

### FGF8 Suppresses Neurotoxic A1 Astrocyte Induction by Inflammatory Cytokines

**Fig. 4:**
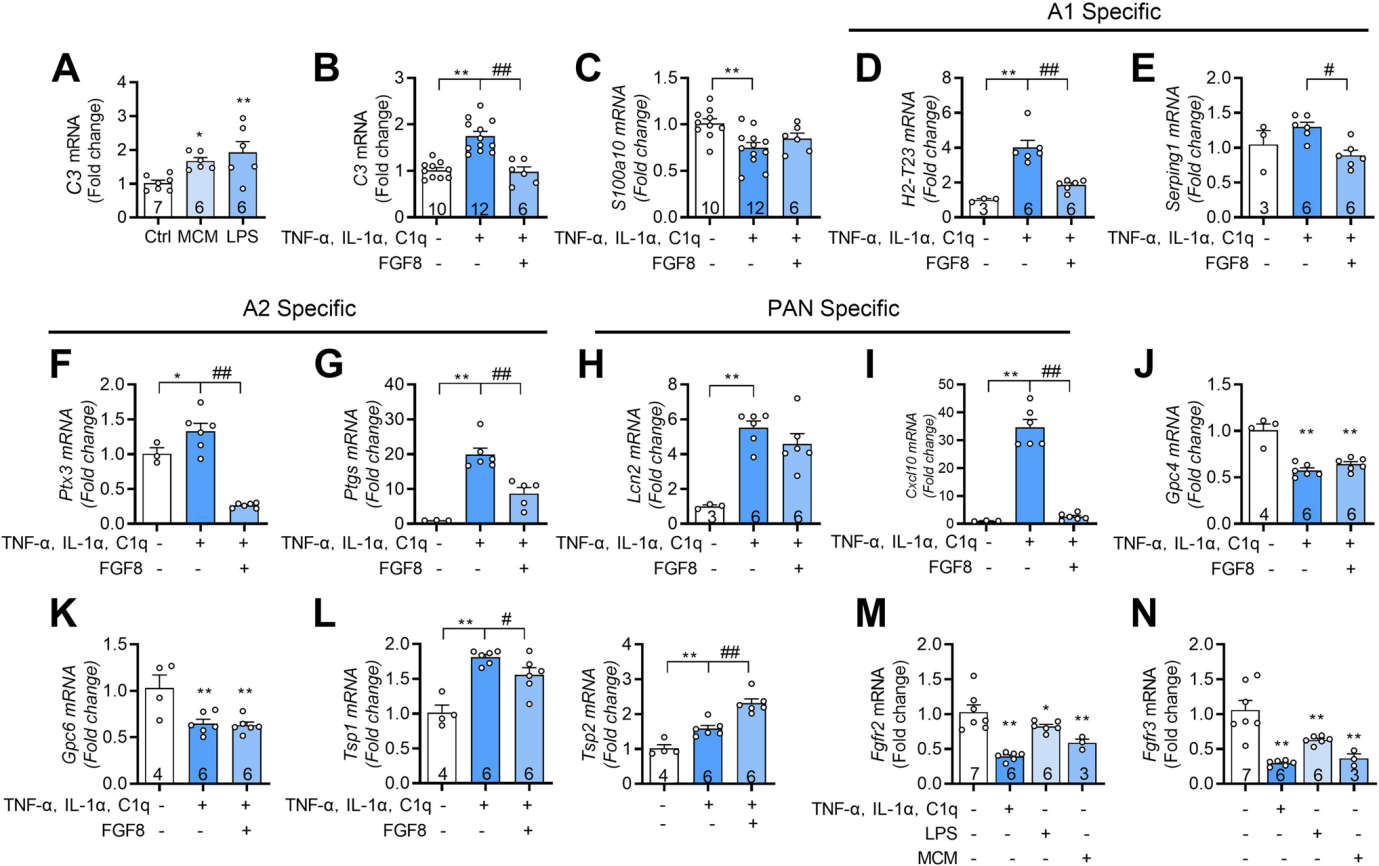
FGF8 alleviates inflammatory induction of neurotoxic astrocyte states in vitro. **A** Relative mRNA expression of *C3* in isolated astrocytes treated with normal culture medium, LPS, or MCM, as measured by qRT-PCR. **B**–**I** Expression of reactive astrocyte markers in astrocytes treated with IL-1α, TNF-α, and C1q, with or without FGF8 co-treatment. A1-specific transcripts: *C3* (**B**), *H2-T23* (**D**), and *Serping1* (**E**); A2-specific transcripts: *S100a10* (**C**), *Ptx3* (**F**), and *Ptgs* (**G**); pan-reactive transcripts: *Lcn2* (**H**) and *Cxcl10* (**I**). **J–L** Relative mRNA expression of synaptogenic genes *Gpc4* (**J**), *Gpc6* (**K**), *Tsp1,* and *Tsp2* (**L**) in astrocytes under the same treatment conditions. **M**, **N** Relative mRNA expression of *Fgfr2* (**M**) and *Fgfr3* (**N**) in isolated astrocytes treated with normal culture medium, LPS, or MCM, as measured by qRT-PCR. *n* = 6–12 petri dishes (**A–C**) and *n* = 3–7 petri dishes (**M**, **N**) derived from pooled spinal cords of 6 mice per preparation; *n* = 3–6 petri dishes (**D–I**) and *n* = 4–6 petri dishes (**J–L**) from pooled spinal cords of 12 mice per preparation. Statistics by one-way ANOVA by *post hoc* Holm-Sidak test (**A–N**). *P < 0.05, **P < 0.01. #*P* < 0.05, ##*P* < 0.01. Data are presented as mean ± SEM. LPS, lipopolysaccharide. MCM, microglia-conditioned media.

Neurotoxic A1 astrocytes can be induced *in vitro* by exposure to inflammatory stimuli such as LPS, MCM, or a combination of IL-1α, TNF-α, and C1q, which mimic the pro-inflammatory milieu observed in neurodegenerative [7, 13–15] and neuropathic pain conditions [17–19]. Primary astrocytes exposed to LPS, MCM, or IL-1α/TNF-α/C1q for 24 h exhibited robust upregulation of C3, a representative A1-specific transcript, thereby establishing a reliable in vitro model for neurotoxic astrocyte activation (Fig. 4A, B).

Given that activated microglia release cytokines capable of triggering A1 astrocyte induction, we next assessed the regulatory effects of FGF8 on astrocytic reactivity. We assessed its impact on the expression of A1-, A2-, and pan-reactive astrocyte markers under inflammatory stimulation in vitro. Transcript analysis showed that either pre-treatment with FGF8 significantly reduced the expression of A1-specific (*C3*, *H2-T23*, *Serping1*), A2-specific (*Ptx3*, *Ptgs*), and pan-reactive (*Cxcl10*) markers (Fig. 4B, D–G, I), indicating that both factors attenuate inflammatory astrocyte activation. To evaluate whether FGF8 affects astrocyte-mediated synaptic support, we examined genes involved in excitatory synaptogenesis. Astrocytes promote excitatory synaptogenesis through glypicans (*Gpc4* and *Gpc6*) [35] and thrombospondins (*Tsp1*and *Tsp2*) [36], which are essential for maintaining synaptic connectivity. Treatment with IL-1α, TNF-α, and C1q significantly reduced *Gpc4* and *Gpc6* expression, while increasing *Tsp1* and *Tsp2* levels, indicating a disruption in synaptogenic function (Fig. 4J–L). Notably, FGF8 treatment did not alter *Gpc4* or *Gpc6* expression but decreased *Tsp1* and increased *Tsp2* levels, suggesting a selective modulation of thrombospondin-related synaptogenic pathways (Fig. 4J–L). Under acute inflammatory conditions in vitro, cytokine-treated astrocytes exhibited reduced expression of both *Fgfr2* and *Fgfr3* compared to non-reactive controls (Fig. 4M, N).

### FGF8 Selectively Enhances Inhibitory Synaptic Transmission and Regulates Synaptogenic Gene Expression

**Fig. 5:**
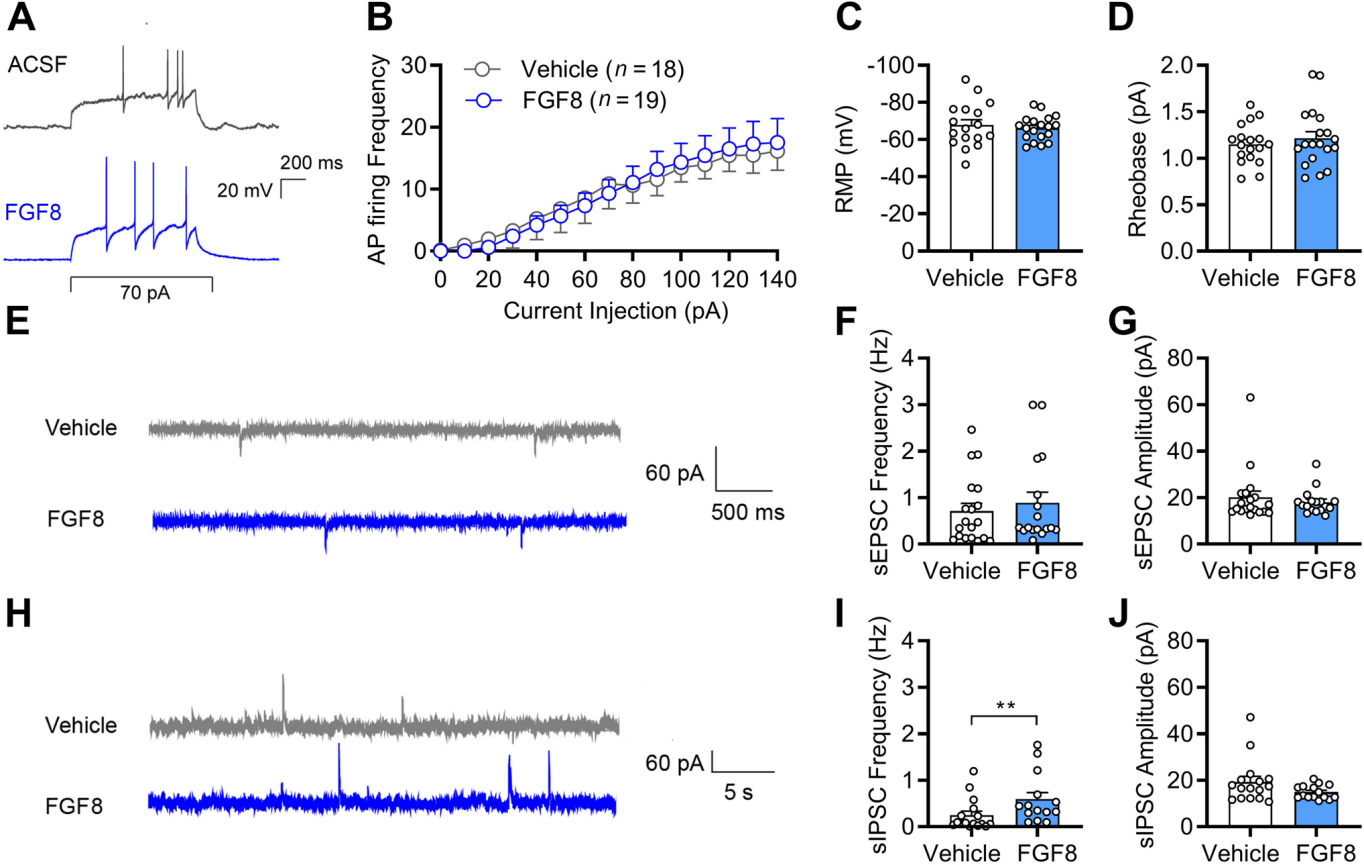
Central action of FGF8 enhances inhibitory synaptic transmission in the spinal cord. **A**–**D** Representative firing traces (**A**) and quantitative statistics showing no significant changes in firing frequencies (**B**), RMP (**C**), or rheobase current (**D**) in lamina II neurons following perfusion with FGF8 (500 ng/mL). **E**–**G** Representative traces of sEPSCs of lamina II neurons (**E**) and quantitative statistics of sEPSC frequency (**F**) and amplitude (**G**). **H**–**J** Representative traces of sIPSCs of lamina II neurons (**H**) and quantitative statistics of sIPSC frequency (**I**) and amplitude (**J**). *n* = 18–19 neurons (**B**–**D**) and *n* = 15–19 neurons (**F**, **G**, **I**, **J**) from 3 mice. Statistics by two-way ANOVA followed by *post hoc* Holm-Sidak test (**B**), two-tailed Student’s *t*-test (**C**, **D**), and Mann-Whitney U test (**F**, **G**, **I**, **J**).**P < 0.01. Data are presented as mean ± SEM. AP, action potential. RMP, resting membrane potential. sEPSC, spontaneous excitatory postsynaptic current. sIPSC, spontaneous inhibitory postsynaptic current.

To investigate the synaptic mechanisms underlying the analgesic effects of FGF8, we first evaluated its impact on synaptic function. Whole-cell patch-clamp recordings were performed on lamina II neurons acutely isolated from SNI mice. FGF8 treatment did not significantly alter intrinsic neuronal excitability (Fig. 5A–D) or the frequency or amplitude of spontaneous excitatory postsynaptic currents (sEPSCs; Fig. 5E–G). In contrast, FGF8 significantly increased the frequency of spontaneous inhibitory postsynaptic currents (sIPSCs) without affecting their amplitude (Fig. 5H–J), indicating a selective facilitation of inhibitory synaptic transmission.

To assess whether this functional enhancement is associated with structural synaptic remodeling, we examined excitatory synaptic markers in the SDH. Immunohistochemistry revealed a marked upregulation of PSD95 and VGLUT2 following SNI. However, intrathecal FGF8 treatment failed to reverse these changes (Fig. S6A–C), suggesting that its analgesic effects are not mediated by restoring excitatory synaptic architecture.

To further explore the molecular correlates of FGF8-induced synaptic modulation, we examined astrocyte-derived synaptogenic genes. In the SNI model, intrathecal FGF8 administration increased *Tsp1* and *Gpc6* expression, while *Tsp2* and *Gpc4* showed no significant changes (Fig. S6D–G). These changes were observed alongside enhanced inhibitory synaptic transmission, without affecting excitatory synaptic markers.

### FGF8 Modulates Astrocytic Responses via FGFR3 Signaling under Microglia-Primed Neuroinflammation

**Fig. 6:**
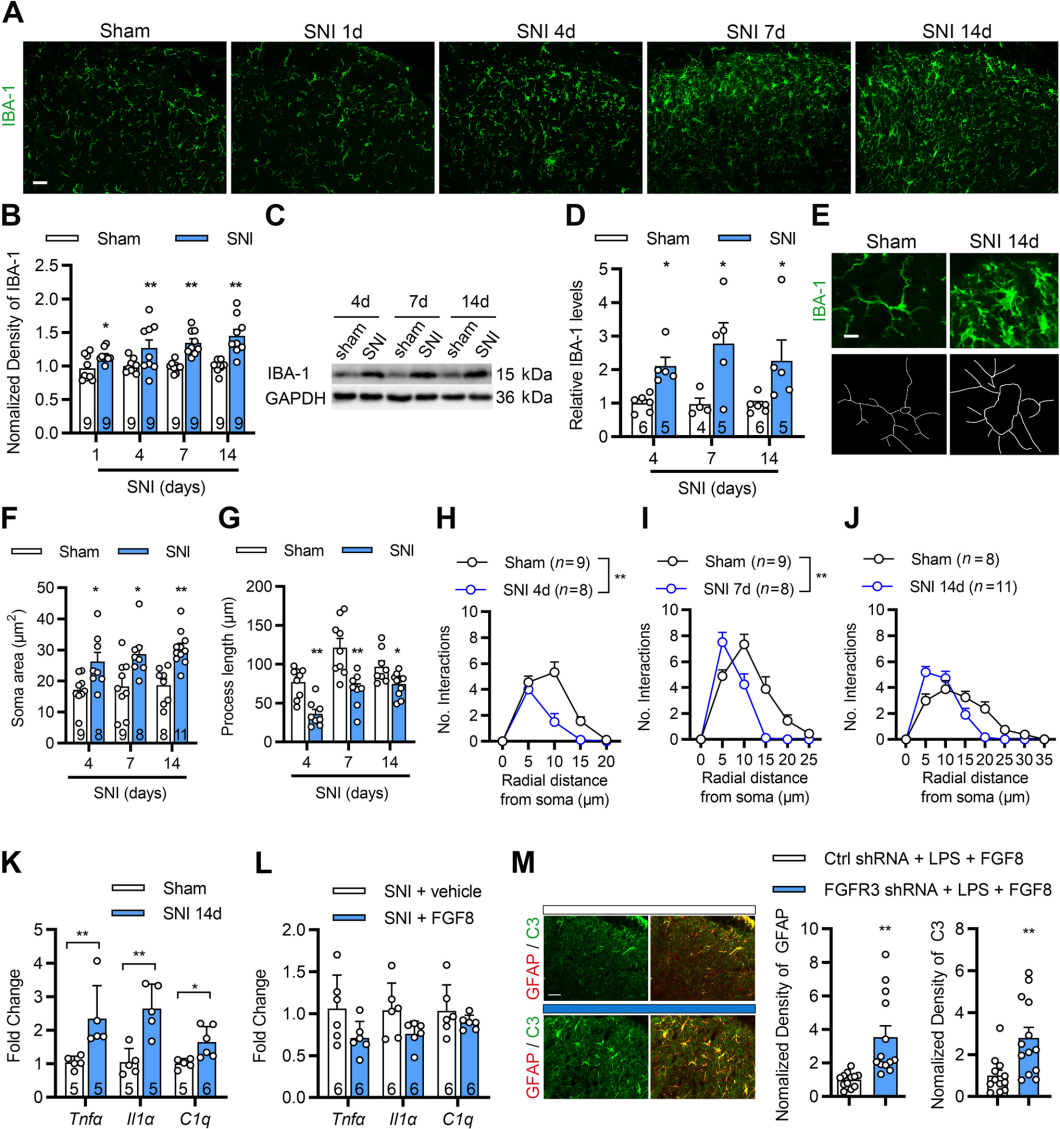
SNI induces persistent microglial activation in the SDH. **A**, **B** Representative immunofluorescence images (**A**) and quantification (**B**) of IBA-1 staining in the ipsilateral SDH from day 1 to day 14 post-SNI. **C**, **D** Representative Western blot images (**C**) and quantification (**D**) of IBA-1 protein levels in the L2–L5 spinal cord segments from day 4 to day 14 post-SNI. **E** Representative skeletonized images of individual IBA-1⁺ microglia from sham and SNI mice. **F**, **G** Quantification of microglial morphology on days 4, 7, and 14 post-SNI, showing a significant increase in soma area (**F**) and a decrease in process length (**G**) compared to sham controls. **H**–**J** Sholl analysis demonstrates reduced microglial process complexity in SNI mice compared to sham controls. *n* = 9 slices. **K** qRT-PCR analysis of *Tnf-α*, *Il-1α*, and *C1q* in the ipsilateral SDH from sham and SNI mice on day 14 (*n* = 5–6 mice). **L** qRT-PCR analysis of *Tnf-α*, *Il-1α*, and *C1q* in spinal cord tissue from SNI mice treated with FGF8 or vehicle on day 14 (*n* = 6 per group). **M** Representative images of C3 and GFAP co-localization and quantification of C3 and GFAP in the SDH of control and FGFR3 shRNA mice following i.t. LPS administration. *n* = 9 slices (**B**), *n* = 8–11 cells (**F**–**J**) from 3 mice; *n* = 4–6 mice (**D**, **K**, **L**); *n* = 12-13 slices (**M**) from 3 mice. Statistics by two-way ANOVA followed by *post hoc* Holm-Sidak test (**B**, **D**, **F**, **G**). Scale bar: 25 μm (**A**, **E**, **M**). Statistics by two-way ANOVA (**H-I**), two-tailed Student’s *t*-test (**K**, **L**), and Mann-Whitney U test (**M**). *P < 0.05, **P < 0.01. Data are presented as mean ± SEM.

Given the close functional interplay between microglia and astrocytes in the CNS, and the well-established role of microglia-derived cytokines in driving astrocytic reactivity [17, 37], we next examined microglial responses in the SDH following SNI. Immunofluorescent staining for ionized calcium-binding adapter molecule 1 (IBA-1), a microglial marker, revealed a marked increase in fluorescence intensity in the ipsilateral SDH post-SNI (Fig. 6A, B), accompanied by elevated IBA-1 protein levels as confirmed by Western blotting (Fig. 6C, D). Morphological features of activation, including soma hypertrophy and process retraction, were also evident at days 4, 7, and 14 post-injury (Fig. 6E–J).

To determine whether this microgliosis involved proliferative activity—a hallmark of microglial expansion after peripheral nerve injury [38], we employed 5-bromo-2’-deoxyuridine (BrdU) assays (100 mg/kg, i.p., twice daily). BrdU is a thymidine analog incorporated into newly synthesized DNA during the S-phase of cell division and is widely used to assess glial proliferation in models of neuropathic pain [38–40]. A marked increase in IBA-1^+^BrdU^+^ microglia was observed in the ipsilateral SDH across all examined time points (days 4, 7, and 14) compared to the sham mice (Fig. S7A). Quantitative analysis showed that IBA-1^+^BrdU^+^ cells comprised 59.2%–66.5% of the total BrdU^+^ mitotic population (Fig. S7D, E), indicating that microglia constitute the dominant proliferative glial subset following SNI. In contrast, SOX9^+^BrdU^+^ astrocytes accounted for only a minor fraction of BrdU^+^ cells (Fig. S7F, G). A significant reduction in SOX9⁺BrdU⁺ cell number was observed at day 4 post-SNI, with no significant changes at later time points. However, the percentage of SOX9⁺BrdU⁺ cells among total BrdU⁺ cells was consistently decreased across all time points (Fig. S7G), indicating a relative suppression of astrocytic proliferation within the proliferating glial population.

We next investigated whether FGF8 modulates neuroinflammation in the SDH following SNI. In line with the observed microglial activation (Fig. 6A–J), SNI induced a marked upregulation of pro-inflammatory cytokines, including *Tnf-α*,* Il-1α*, and *C1q*, in the ipsilateral SDH (Fig. 6K). Treatment with FGF8 showed a trend toward reducing *Tnf-α* and* Il-1α* levels (Fig. 6L, *P* = 0.08), though not statistically significant under the current experimental conditions. To further elucidate the role of astrocytic FGFR3 under microglia-driven inflammatory conditions, we employed a LPS-induced neuroinflammation model previously established to selectively activate microglia [33]. Conditional knockdown of FGFR3 in astrocytes significantly increased the proportion of C3⁺GFAP⁺ astrocytes compared to controls, indicating enhanced A1-like neurotoxic reactivity (Fig. 6M). These results suggest that astrocytic FGFR3 may serve as a critical modulator of astrocyte phenotypes in microglia-primed neuroinflammation.

## Discussion

Astrocytes, once considered a relatively homogeneous population, are now recognized as highly heterogeneous cells with distinct morphological, molecular, and functional properties across brain regions and physiological states [2–6]. Recent advances in single-cell transcriptomics have revealed diverse astrocyte subtypes in the adult cortex and hippocampus, including region-specific populations within cortical layers [4, 5]. This heterogeneity is further amplified under pathological conditions such as infection, ischemic injury, or neuroinflammation, where astrocytes undergo context-dependent reactive transformations characterized by dynamic transcriptional and functional changes [2, 6, 8, 41]. Importantly, these reactive phenotypes are not uniform. Transcriptomic analyses in models of LPS challenge, ischemic stroke, and autoimmune encephalomyelitis (EAE) have shown that astrocyte responses are shaped by both the type of insult and the affected region [8, 41], underscoring the need for therapeutic strategies that account for this complexity. Understanding how specific reactive states are initiated and maintained is essential for identifying precise molecular targets in CNS disorders, including chronic pain. In our previous work, we demonstrated that astrocytes in the SDH are activated and contribute to central sensitization across various chronic pain models, including inflammatory pain, chemotherapy-induced neuropathy, and cancer-induced bone pain [31, 42–44]. Building on this foundation, the current study focuses on characterizing astrocyte phenotype transitions in a neuropathic pain model and identifying potential molecular modulators of these changes. We demonstrate that astrocytes in the SDH adopt a reactive state following SNI, characterized by elevated GFAP expression and transcriptomic reprogramming. While the overall number of GFAP^+^ or SOX9^+^ astrocytes remained unchanged, there was a significant increase in both C3^+^GFAP^+^ and S100A10^+^GFAP^+^ astrocytes, indicative of a phenotypic transition. Transcriptomic profiling further confirmed the enrichment of A1-specific and pan-reactive astrocyte signatures post-SNI. These results are consistent with findings from other neuropathic pain models [17–19, 45]. Notably, the pronounced elevation of S100A10⁺GFAP⁺ astrocytes may reflect a potential self-protective mechanism, suggesting that distinct astrocytic subpopulations play divergent roles in neuropathic pain progression. Importantly, FGF8 treatment selectively suppressed the induction of C3^+^GFAP^+^ astrocytes without significantly altering the proportion of S100A10^+^ cells under pathological pain conditions. These findings indicate that FGF8 preferentially attenuates neurotoxic A1-like reactivity while sparing potentially beneficial A2-associated responses. Such context-specific modulation may represent a favorable mechanism for restoring glial homeostasis while preserving protective astrocytic functions.

Microglia-derived cytokines, including IL-1α, TNF-α, and C1q, are well-established inducers of neurotoxic A1 astrocytes [7, 46]. Previous strategies to suppress A1 astrocyte formation have mainly targeted upstream microglial signaling, such as through triple knockouts of *Il-1α*, *Tnf-α*, and *C1q* or pharmacological interventions including GLP-1R agonists[15], PKCδ inhibitors [47], and NLRP3 inhibitors [48]. In contrast, targeting astrocytes directly may offer a more efficient and precise approach to restoring glial homeostasis. Indeed, modulating astrocyte-intrinsic regulators—such as TMEM164 [13], TDP-43 [49], or the P2Y1 receptor [50]—has been shown to alter reactive phenotypes and influence behavioral outcomes. In the present study, we identify FGF8 as an effective modulator of spinal astrocytic reactivity under neuropathic pain conditions. FGF8 significantly reduced the expression of A1-specific transcripts both *in vitro* and *in vivo.* Notably, intrathecal administration of FGF8 significantly alleviated mechanical allodynia in SNI mice, highlighting its therapeutic relevance.

Our findings suggest that FGF8 modulates synaptic function via mechanisms distinct from structural remodeling. Although SNI induced prominent structural changes in the dorsal horn, including upregulation of PSD95 and VGLUT2, these markers remained elevated following FGF8 treatment, indicating limited impact on excitatory synaptic architecture. In contrast, whole-cell patch-clamp recordings from lamina II neurons revealed a selective increase in sIPSC frequency after FGF8 administration, with no alteration in sEPSCs or intrinsic neuronal excitability, pointing toward a functional enhancement of inhibitory drive. Mechanistically, this was accompanied by a selective upregulation of astrocyte-derived synaptogenic factors *Tsp1* and *Gpc6*, while *Tsp2* and *Gpc4* remained unchanged. Although these genes are traditionally implicated in excitatory synapse formation, their upregulation in this context may reflect a compensatory or circuit-specific role in inhibitory network support. Mechanistically, we identify the FGF8-FGFR3 signaling axis as a critical regulator of astrocytic responses and inhibitory synaptic modulation under neuropathic conditions. Single-nucleus RNA sequencing from the adult mouse spinal cord confirmed FGFR3 enrichment in astrocytes, supporting its potential as a key mediator of FGF8 effects. Immunofluorescence analysis further demonstrated an increase in FGFR3⁺GFAP⁺ astrocytes following SNI. Consistently, bulk qPCR analysis of SDH tissue confirmed a robust upregulation of *Fgfr3* after SNI. In contrast, transcriptomic profiling of ACSA-2-sorted astrocytes showed no significant change in *Fgfr3*, which may reflect differences in cell-type enrichment and detection sensitivity. Supporting this, triple staining confirmed FGFR3 expression in C3^+^GFAP^+^ A1-like astrocytes as well as some GFAP⁻ cells, suggesting that FGFR3 may be expressed in broader cellular populations. These findings underscore the importance of integrating complementary approaches when evaluating receptor expression in heterogeneous tissues and highlight the need for future studies to delineate the distribution and functional roles of FGFR3 across diverse glial subtypes.

Functionally, conditional deletion of *Fgfr3* in astrocytes abolished the analgesic effects of FGF8 in SNI mice, confirming that astrocytic FGFR3 is essential for FGF8-mediated modulation of neuropathic pain. In contrast, pharmacological blockade of FGFR2 failed to affect FGF8-induced analgesia, further supporting the conclusion that FGFR3, rather than FGFR2, mediates the primary signaling pathway by which FGF8 regulates astrocytic reactivity and pain sensitivity. Taken together, these findings establish astrocytic FGFR3 as a functionally relevant mediator of FGF8 effects and highlight its dual role in limiting maladaptive astrocytic reactivity and enhancing inhibitory synaptic transmission, thereby contributing to pain relief.

To further explore microglia–astrocyte crosstalk, we employed an LPS-induced inflammatory model previously shown to activate microglia [33]. Conditional knockdown of FGFR3 in astrocytes markedly increased the proportion of C3⁺GFAP⁺ astrocytes under LPS challenge, indicating enhanced neurotoxic A1-like reactivity. These findings suggest that astrocytic FGFR3 acts as a critical gatekeeper of reactive phenotype conversion, mediating how astrocytes respond to microglial-derived inflammatory cues. Together, these results reveal a dual role of FGF8 in attenuating microglia-driven astrocytic neurotoxicity and highlight its potential for selectively targeting maladaptive glial responses in neuropathic pain.

Previous studies have implicated FGFR3 in regulating astrocytic morphology and reactivity [27, 32]. Together with our data, these findings support a role for the FGF8-FGFR3 axis in modulating astrocyte phenotype transitions, particularly in suppressing neurotoxic A1 reactivity while sparing or supporting protective A2-associated responses. Despite these promising findings, several limitations warrant further investigation. While FGF8 significantly enhanced inhibitory synaptic transmission in lamina II, we did not directly evaluate structural remodeling of synapses. Moreover, it remains unclear whether these effects are mediated primarily through astrocyte-intrinsic mechanisms or involve crosstalk with defined neuronal populations. Future studies using cell-type-specific manipulations and high-resolution imaging will be essential to clarify these circuit-level interactions underlying FGF8-FGFR3 signaling in neuropathic pain. Moreover, the precise downstream mechanisms by which FGF8-FGFR3 signaling exerts its modulatory effects remain undefined. At the molecular level, FGF8 treatment increased PLCE1 expression, suggesting potential engagement of Ras signaling downstream of FGFR3 activation. Interestingly, transcriptomic enrichment analysis from previous studies identified *Fgfr3* and *Plce1* as co-associated components within the Ras signaling pathway [51]. Further functional studies will be required to clarify the causal role of PLCE1 in mediating FGFR3-driven astrocytic responses. Moreover, although astrocyte–microglia interactions have been implicated in shaping glial reactivity [52], the extent to which FGF8 influences microglial–astrocytic signaling in neuropathic pain remains unclear. Our current findings under LPS stimulation suggest a partial suppression of microglia-driven astrocytic activation by FGF8, but whether similar mechanisms occur in the SNI model warrants further exploration.

In conclusion, our study highlights the critical role of astrocytic phenotypic transitions in the pathogenesis of neuropathic pain and identifies the FGF8-FGFR3 signaling axis as a promising modulator of astrocytic reactivity. By preferentially suppressing neurotoxic A1 astrocyte induction via FGFR3 and ameliorating pain hypersensitivity, FGF8 emerges as a promising candidate therapeutic intervention. Future studies are warranted to elucidate downstream mechanisms, assess long-term efficacy, and develop translational strategies for FGF8–FGFR3-based therapies targeting chronic pain.

## Supplementary Information

Below is the link to the electronic supplementary material.

Supplementary file1 (PDF 1715 KB)
