# Supplementary material for: Fibroblast Growth Factor 8 Suppresses Neurotoxic Astrocytes and Alleviates Neuropathic Pain via Spinal FGFR3 Signaling
Source: Neurosci Bull. 2025 Oct 30;41(12):2218–32. doi: 10.1007/s12264-025-01533-x (PMC12698887; doi:10.1007/s12264-025-01533-x)
Supplement: Supplementary file 1 — Supplementary file1 (PDF 1715 KB) [file 12264_2025_1533_MOESM1_ESM.pdf]

Supporting Figures (Fig. S1-S7)

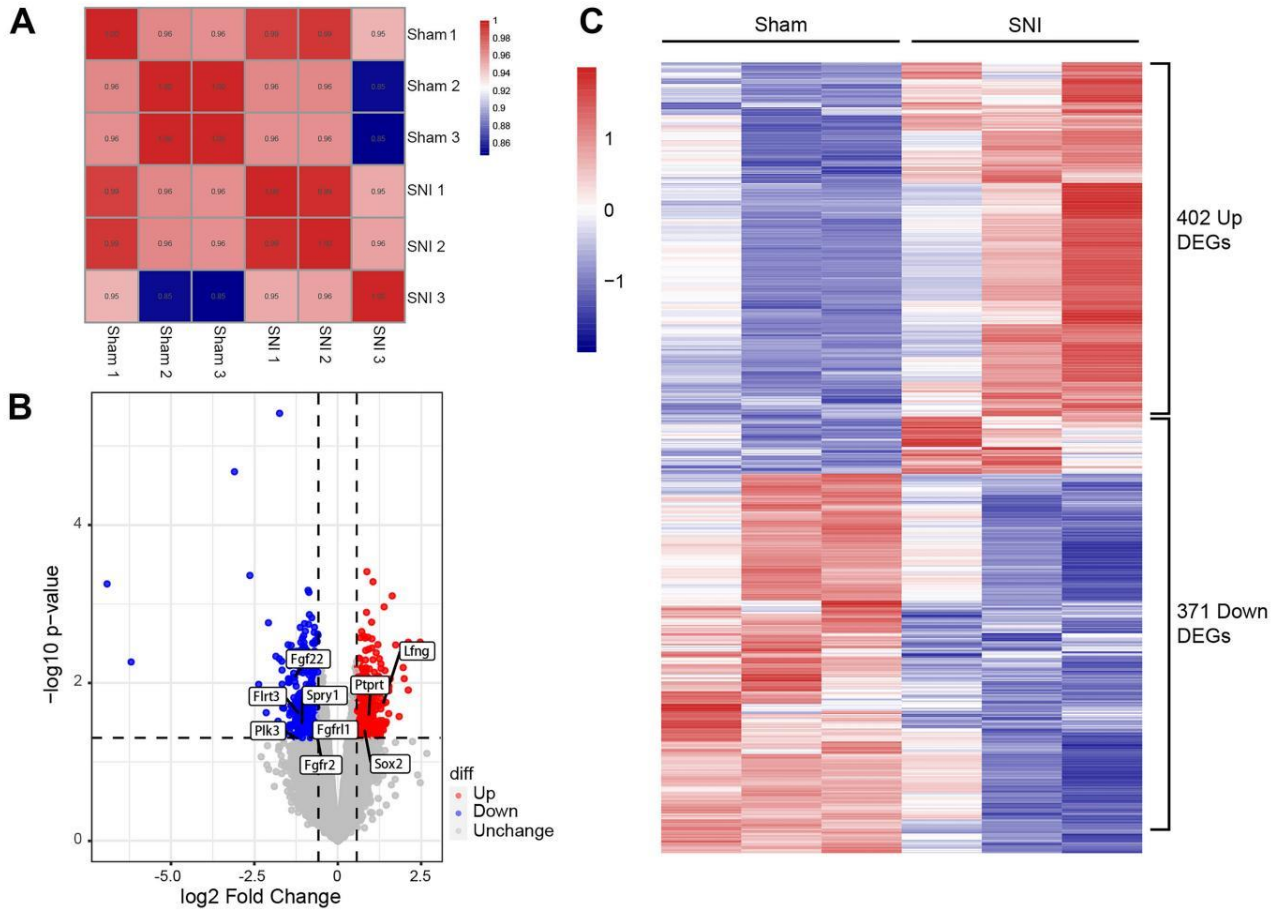

Fig. S1 Transcriptomic profiling of spinal astrocytes reveals differential gene expression in the SNI model. (**A**) Correlation heatmap showing the similarity of gene expression profiles between sham and SNI samples. (**B**) Volcano plot displaying DEGs in SNI samples compared to sham controls. Red: upregulated genes; blue: downregulated genes. (**C**) Heatmap of 773 DEGs (402 upregulated, 371 downregulated), illustrating distinct transcriptomic alterations in the SNI group. DEG cut off: $P < 0.05$ and $|\log_2 FC| > 0.58$. DEGs, differentially expressed genes.

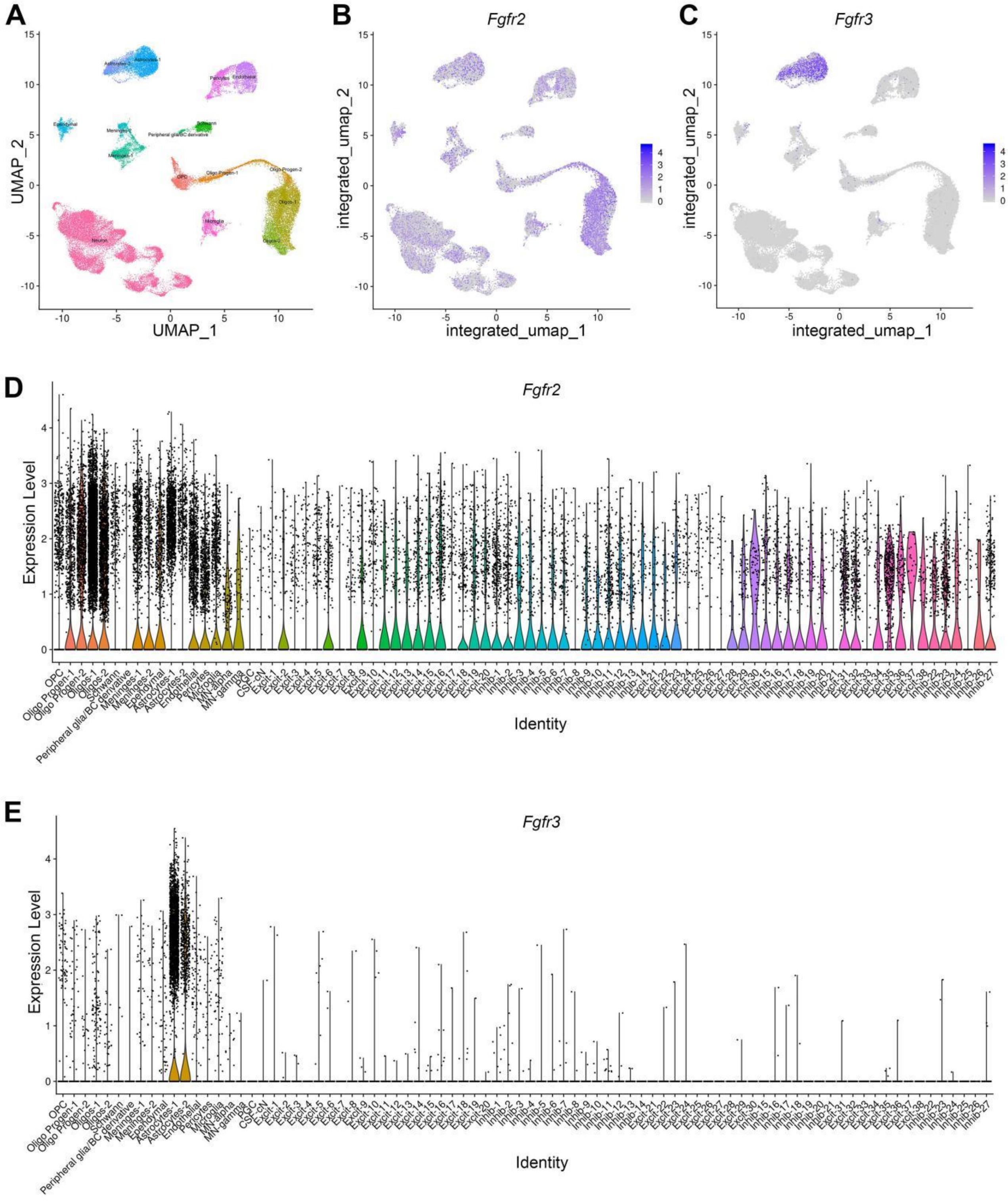

Fig. S2 Single-nucleus RNA sequencing reveals cell-type-specific expression of *Fgfr2* and *Fgfr3* in the mouse spinal cord. (**A**) UMAP plot showing the major cell types identified from snRNA-seq of adult mouse spinal cord. (**B, C**) Feature plots of *Fgfr2* (**B**) and *Fgfr3* (**C**) expression across all clusters.

(**D, E**) Violin plots showing cell type-specific expression levels of *Fgfr2* (**D**) and *Fgfr3* (**E**).

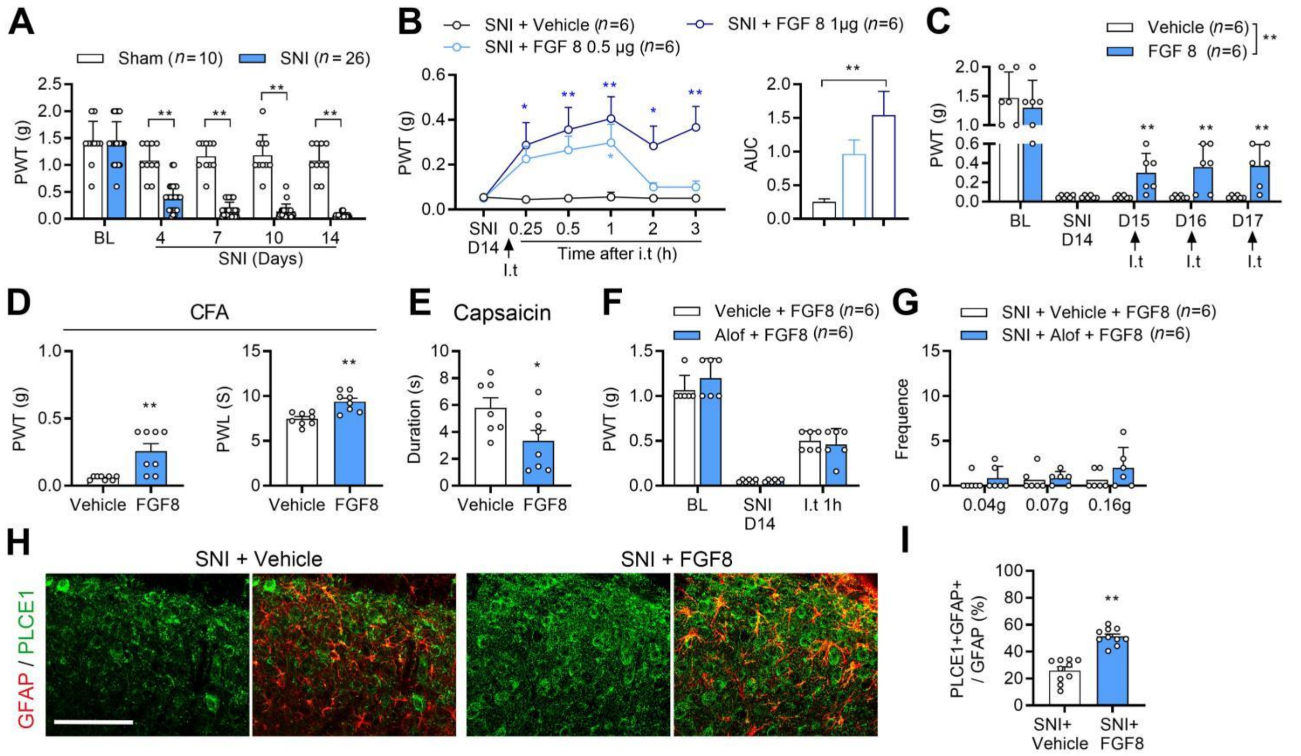

Fig. S3 FGF8 alleviates SNI-induced neuropathic pain, CFA-induced inflammation pain, and capsaicin-induced spontaneous pain. (**A**) SNI induces significant mechanical allodynia in the ipsilateral hindpaw. (**B**) Single i.t. injection of FGF8 (1 μg in 5 μL) on day 14 post-SNI significantly increased the paw withdrawal threshold and reduced response frequency to *von* Frey stimulation. (**C**) Daily FGF8 injection for three consecutive days during day 15 to day 17 post-SNI maintained stable analgesia. (**D, E**) A single i.t. injection of FGF8 alleviated mechanical allodynia and thermal hyperalgesia induced by CFA (**D**), and spontaneous pain responses induced by capsaicin (**E**). (**F, G**) The FGFR2-selective antagonist Alofanib (Alof, 10 μg in 5 μL) failed to reverse the analgesic effects of i.t. FGF8 injection on day 14 post-SNI. *n* =10-26 mice (**A**), *n* =6-8 mice [(**B**), (**C**), (**D**), (**E**), (**F**), (**G**)], and *n* = 10 slices (**I**) from 3 mice. Scale bar: 25 μm (**H**). Statistics by two-way ANOVA followed

by *post hoc* Holm-Sidak test [(**A**), (**B** left), (**C**), (**F**), (**G**)], One-way ANOVA by *post hoc* Holm-Sidak test (**B** right), Mann-Whitney U test (**D**), and two-tailed Student's *t*-test (**E**). *$P < 0.05$. **$P < 0.01$. Data are presented as mean ± SEM, unless otherwise specified. Sample sizes are indicated in the bars or figure panels. AUC, area under the curve. I.t, intrathecal injection.

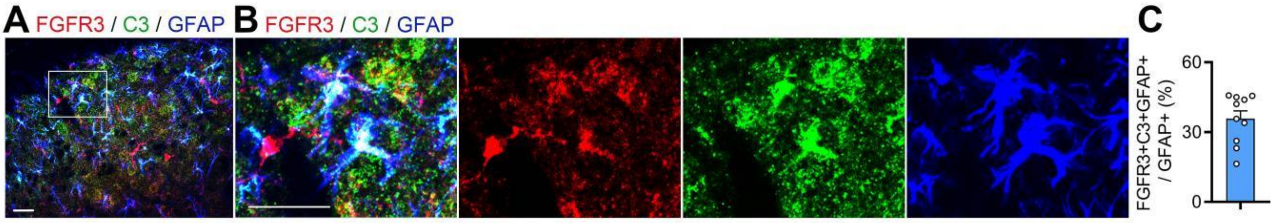

Fig. S4 FGFR3 co-localized with C3-positive astrocytes in the spinal dorsal horn. (**A-C**) Representative images showing co-localization of FGFR3 with C3 and GFAP in the SDH, with $FGFR3^{+}C3^{+}GFAP^{+}$ cells accounting for approximately 35.8% of the total $GFAP^{+}$ population. $n = 10$ slices from 3 mice. Scale bar: 20 μm. Data are presented as mean ± SEM.

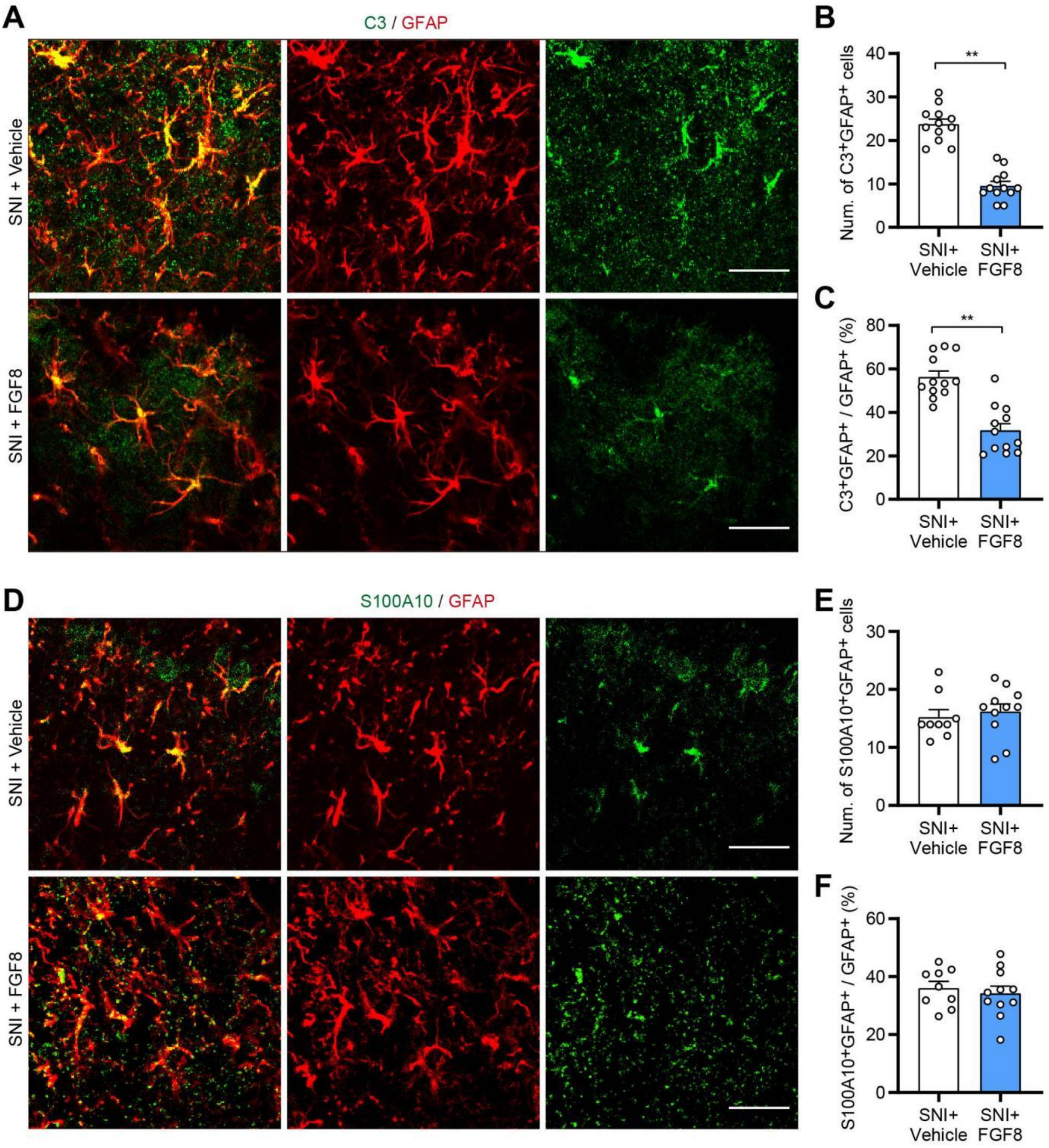

Fig. S5 FGF8 treatment significantly reduced the proportion of $C3^+$ astrocytes in SNI mice. (**A-C**) Immunofluorescence staining and quantitative data showing FGF8 treatment significantly reduced the number and proportion of $C3^+$ astrocytes. (**D-F**) Immunofluorescence staining and quantitative data showing FGF8 treatment did not change the number and proportion of $C3^+$ astrocytes. n = 9-12 slices

from 3 mice [(**B**), (**C**), (**E**), (**F**)]. Scale bar: 20 μm. Statistics by two-tailed Student's *t*-test [(**B**), (**C**), (**F**)] and Mann-Whitney U test (**E**). $^{**}P < 0.01$. Data are presented as mean ± SEM.

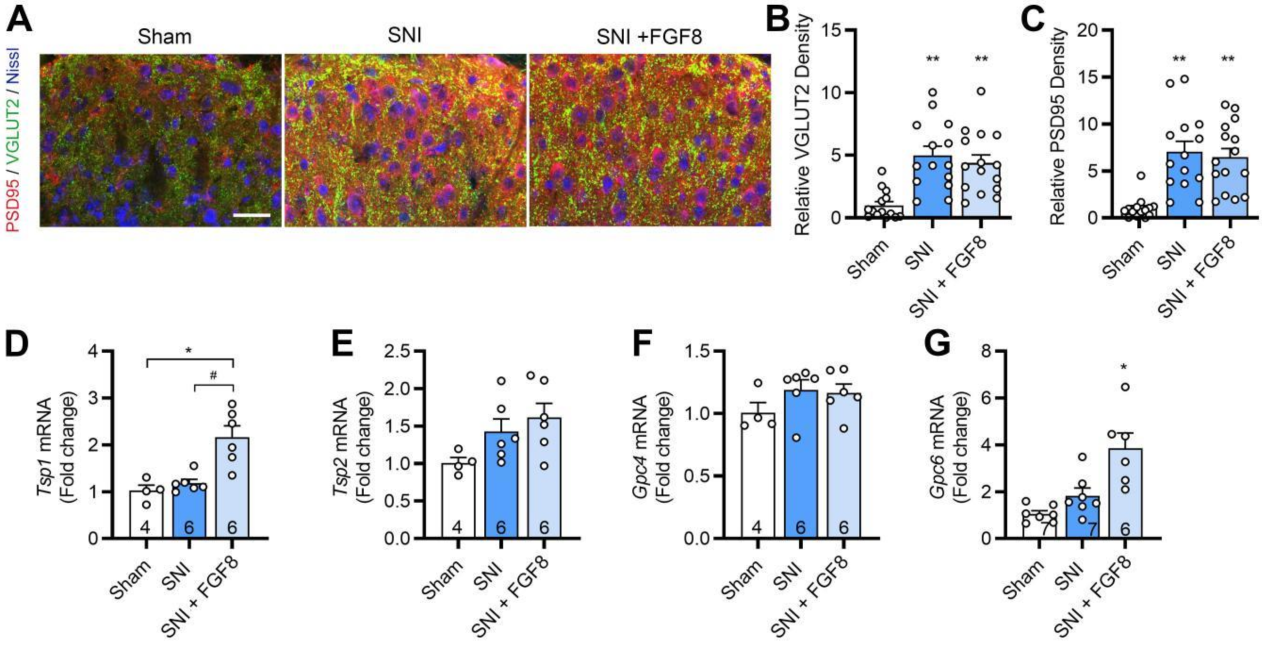

Fig. S6 Evaluation of synaptic markers and astrocyte-associated synaptogenic gene expression after SNI and FGF8 treatment. (**A-C**) Representative images and quantification showing the expression of PSD95 (the postsynaptic marker) and VGLUT2 (the presynaptic marker) in SDH from sham and SNI mice with or without FGF8 treatment. (**D-G**) Relative mRNA expression of astrocyte-associated synaptogenic genes *Tsp1* (**D**), *Tsp2* (**E**), *Gpc4* (**F**), and *Gpc6* (**G**) in spinal cord tissue from sham and SNI mice with or without FGF8 treatment. n = 14-15 slices from 3 mice [(**B**), (**C**)] and n = 4-7 mice (**D-G**). Scale bar, 25 μm (**A**). Statistics by Kruskal-Wallis test [(**B**), (**C**)], and one-way ANOVA followed by *post hoc* Holm-Sidak test (**D-G**). $^{*}P < 0.05$. $^{**}P < 0.01$. $^{\#}P < 0.05$. Data are presented as mean ± SEM. Sample sizes are indicated in the bars or figure panels.

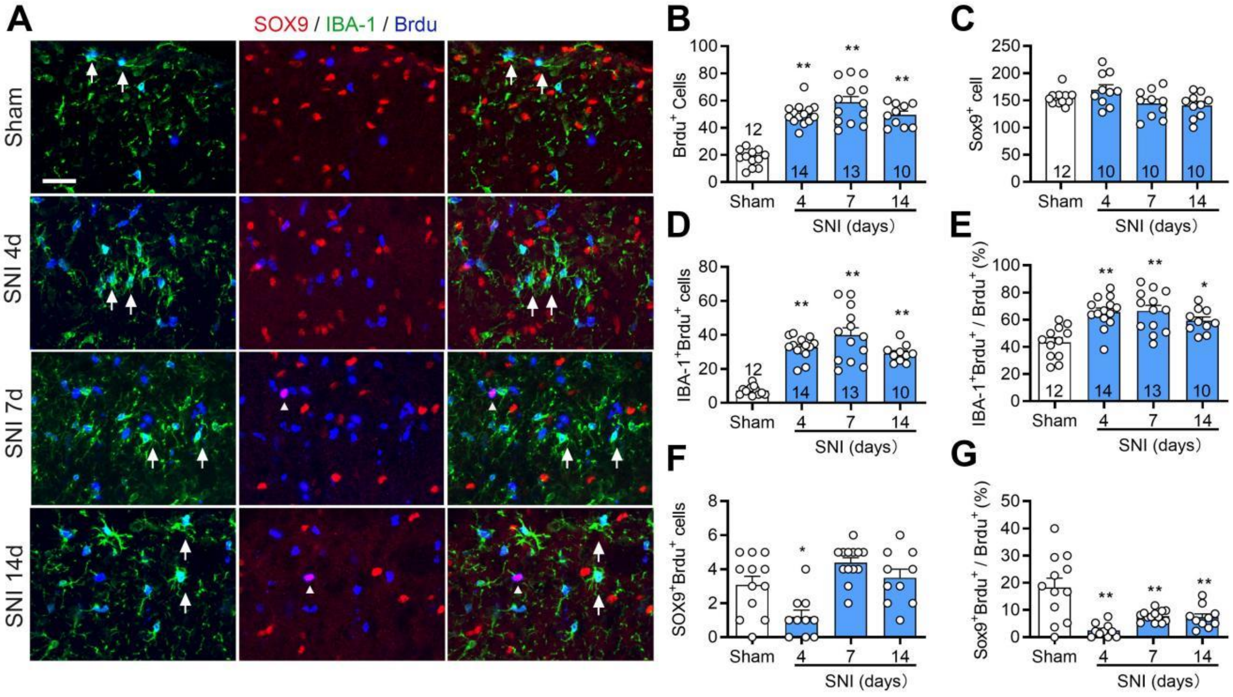

Fig. S7 SNI promotes microglial proliferation in the SDH. (**A**) Triple immunofluorescence staining showing co-localization of $BrdU^+$ (blue), IBA-1^+ (green), and $SOX9^+$ (red, a nuclear marker for astrocytes) cells in the SDH after SNI. Arrows indicate the typical cell. (**B-D**) Quantification of $BrdU^+$ cells (B), $SOX9^+$ astrocytes (**C**), and IBA-$1^+$$BrdU^+$ proliferating microglia (**D**) on days 4, 7, and 14 post-SNI. (**E-G**) Quantification of the percentage of IBA-$1^+$$BrdU^+$ cells among total $BrdU^+$ cells (**E**), the total number of $SOX9^+$$BrdU^+$ cells (**F**), and the percentage of $SOX9^+$$BrdU^+$ cells among total BrdU+ cells (**G**). *n* = 10-14 slices from 3 mice (**B-G**). Scale bar, 25 μm (**A**). Statistics by one-way ANOVA by *post hoc* Holm-Sidak test (**B-G**). *$P < 0.05$, **$P < 0.01$. Data are presented as mean ± SEM. Sample sizes are indicated in the bars or figure panels.

Supporting table (Table S1)

Table S1 The key resources and reagents used in the present study

Reagent or Resource	Source	Identifier	
**Antibodies**			
**Immunohistochemistry**			
mouse monoclonal anti-GFAP (1:2000)	Sigma	CAT# G6171	RRID:AB_1840893
goat polyclonal anti-IBA-1 (1:500)	Abcam	CAT# 5076	RRID:AB_2224402
goat polyclonal anti-SOX9 (1:500)	R&D	CAT# AF3075	RRID: AB_2194160
rat monoclonal anti-Brdu (1:1000)	Abcam	CAT# ab6326	RRID: AB_305426
goat polyclonal anti-complement component C3 (1:50)	R&D	CAT# AF2655	RRID: AB_2066622
rabbit polyclonal anti-S100A10 (1:100)	Abcam	CAT# ab187201	N/A
guinea pig anti-VGLUT2 (1:500)	Millipore	Cat# AB2251	RRID: AB_1587626

rabbit polyclonal anti-PSD95	Abcam	CAT# ab18258	RRID: AB_10 106629
NeuroTrace□ Fluorescent Nissl Stains (1:500)	Invitrogen	CAT#N214 79	N/A
Rabbit polyclonal anti-FGFR3 (1:200)	Invitrogen	CAT# PA5-34574	RRID:AB_2551 926
Rabbit polyclonal anti-PLCE1 (1:200)	Thermo	CAT# PA5-100856	RRID:AB_2850 354
Donkey anti-Rat IgG (H+L) Highly Cross-Adsorbed Secondary Antibody, Alexa Fluor™ 488 (1:200)	Invitrogen	CAT# A21208	RRID:AB_2535 794
Donkey Anti-Mouse IgG (H+L) Highly Cross-Adsorbed Secondary Antibody, Alexa Fluor™ 488 (1:200)	Invitrogen	CAT# A-21202	RRID:AB_1416 07
Donkey anti-Mouse IgG (H+L) Highly Cross-Adsorbed Secondary Antibody, Alexa Fluor™ 546 (1:200)	Invitrogen	CAT# A10036	RRID:AB_2534 012
Donkey Anti-Goat IgG (H+L) Cross-Adsorbed Secondary Antibody, Alexa Fluor™ 488 (1:200)	Invitrogen	CAT# A-11055	RRID:AB_1426 72
Donkey Anti-Goat IgG (H+L) Cross-Adsorbed Secondary Antibody, Alexa Fluor™	Invitrogen	CAT# A-11056	RRID:AB_1426 28

546 (1:200)			
Cy2-conjugated AffiniPure Donkey Anti-Guinea Pig IgG (H+L) (1:500)	Jackson ImmunoResearch Labs	CAT# 706-225-148	RRID:AB_2340467
Cy3-conjugated AffiniPure Donkey Anti-Rabbit IgG (H+L) (1:500)	Jackson ImmunoResearch Labs	CAT# 711-165-152	RRID:AB_2340604
Cy5-conjugated AffiniPure Donkey Anti-Rabbit IgG (H+L) (1:500)	Jackson ImmunoResearch Labs	CAT# 711-175-152	RRID:AB_2340607
Cy2-conjugated AffiniPure Donkey Anti-Rabbit IgG (H+L) (1:500)	Jackson ImmunoResearch Labs	CAT# 711-225-152	RRID:AB_2340612
**Western Blotting**			
mouse monoclonal anti-GFAP (1: 20000)	Sigma	Cat# G6171	RRID:AB_1840893
rabbit polyclonal anti-IBA-1 (1: 1000)	Wako Pure Chemical Industries	Cat# 019-19741	RRID:AB_839504
HRP-GAPDH (1: 20000)	Aksomics	Cat# KC-5G5	RRID:AB_2631280
goat polyclonal anti-complement component C3 (1: 1000)	R&D	CAT# AF2655	RRID: AB_2066622
rabbit polyclonal anti-S100A10 (1: 1000)	Abcam	CAT# ab187201	N/A
HRP-conjugated goat anti-rabbit (1: 5000)	Jackson	Cat# 111-035-003	RRID:AB_2313567
HRP-conjugated donkey anti-mouse (1: 1000)	Santa Cruz	Cat# sc-2096	RRID:AB_641168
HRP-conjugated Rabbit Anti-Goat IgG (1: 2000)	Proteintech	Cat# SA00001-4	N/A

**Chemicals, peptides, and recombinant proteins**			
DAPI (1: 20000)	Thermo Fisher Scientific	Cat# D3571	RRID: AB_2307445
IL-1α	Sigma	Cat# I3901	N/A
TNFα	Cell Signaling Technology	Cat# 8902SF	N/A
C1q	Sigma	Cat# I3901	N/A
Lipopolysaccharide	Sigma	CAT # L2880	N/A
Recombinant Human/Mouse FGF-8 Protein (FGF8)	R&D Systems	CAT # 423-F8-025/CF	N/A
Thymidine analog 5-bromo-2'-deoxyuridine (BrdU)	Sigma	Cat# B5002	N/A
Alofanib	Selleck	S8754	N/A
Complete Freund's adjuvant	Sigma	F5881	N/A
Capsaicin	MCE	HY-10448R	N/A
**Bacterial and viral strains**			
AAV- GFAabc1d -EGFP-mi30-shRNA (FGFR3 shRNA) (AAV2/5, titer: 3.88 ×10^{13} V.G./mL)	Sunbio Medical Biotechnology Company, Shanghai, China	N/A	N/A
AAV- GFAabc1d -EGFP-mi30-shRNA (Ctrl shRNA) (AAV2/5, titer: 3.29 ×10^{13} V.G./mL)	Sunbio Medical Biotechnology Company, Shanghai, China	N/A	N/A
**Primers for quantitative real-time PCR**			
Actin Forward: 5'-GGCTGTATTCCCCTCCATCG-3'			All primers were synthesized by Sangon Biotech Co., Ltd.; identifiers not applicable
Actin Reverse: 5'-CCAGTTGGTAACAATGCCATGT-3'			
C3 Forward: 5'-CCAGCTCCCCATTAGCTCTG-3'			
C3			

Reverse: 5’-GCACTTGCCTCTTTAGGAAGTC-3’	(N/A).
S100a10<br>Forward: 5’-CCTCTGGCTGTGGACAAAAT -3’	
S100a10<br>Reverse: 5’-CTGCTCACAAGAAGCAGTGG -3’	
Il-1α<br>Forward: 5’-AAACTTCTGCCTGACGAGCTT -3’	
Il-1α<br>Reverse: 5’-GCACCTTACACCTACCAGAGT -3’	
Tnfα<br>Forward: 5’-CCCTCACACTCAGATCATCTTCT -3’	
Tnfα<br>Reverse: 5’-GCTACGACGTGGGCTACAG -3’	
C1q<br>Forward: 5’-AAAGGCAATCCAGGCAATATCA -3’	
C1q<br>Reverse: 5’-TGGTTCTGGTATGGACTCTCC -3’	
Fgfr2<br>Forward: 5’- CACGACCAAGAAGCCAGACT-3’	
Fgfr2<br>Reverse: 5’- CTCGGCCGAAACTGTTACCT-3’	
Fgfr3<br>Forward: 5’- GTGGTGGCAGCTGTGATACT-3’	
Fgfr3<br>Reverse: 5’- TTAAGCGGGAAGCGAGAGAC-3’	
Gpc4<br>Forward: 5’-CTGGAGGGTCCTTTCAACATT -3’	
Gpc4<br>Reverse: 5’-GACATCAGTAACCAGTCGGTC -3’	
Gpc6<br>Forward: 5’-TAGTCCTGTATTGGCAGCCAC -3’	
Gpc6<br>Reverse: 5’-GGCTAATGTCTATAGCAGGGAA -3’	
Tsp1<br>Forward: 5’-GAAGCAACAAGTGGTGTCAGT -3’	
Tsp1<br>Reverse: 5’-ACAGTCTATGTAGAGTTGAGCCC -3’	
Tsp2<br>Forward: 5’-CCTCAACTACTGGGTAGAAGGC -3’	
Tsp2<br>Reverse: 5’-TGACACTGTCGATAAGATCGCA -3’	

H2-T23<br>Forward: 5'-GGACCGCGAATGACATAGC -3'			
H2-T23<br>Reverse: 5'-GCACCTCAGGGTGACTTCAT -3'			
Serping1<br>Forward: 5'-ACAGCCCCCTCTGAATTCTT -3'			
Serping1<br>Reverse: 5'-GGATGCTCTCCAAGTTGCTC -3'			
H2-D1<br>Forward: 5'-TCCGAGATTGTAAAGCGTGAAGA -3'			
H2-D1<br>Reverse: 5'-ACAGGGCAGTGCAGGGATAG -3'			
Ptx3<br>Forward: 5'-AACAAGCTCTGTTGCCCATT -3'			
Ptx3<br>Reverse: 5'-TCCCAAATGGAACATTGGAT -3'			
Ptgs2<br>Forward: 5'-GCTGTACAAGCAGTGGCAAA -3'			
Ptgs2<br>Reverse: 5'-CCCCAAAGATAGCATCTGGA -3'			
Lcn2<br>Forward: 5'-CCAGTTCGCCATGGTATTTT -3'			
Lcn2<br>Reverse: 5'-CACACTCACCACCCATTCAG -3'			
Cxcl10<br>Forward: 5'-CCCACGTGTTGAGATCATTG -3'			
Cxcl10<br>Reverse: 5'-CACTGGGTAAAGGGGAGTGA -3'			
Serpina3n<br>Forward: 5'-CCTGGAGGATGTCCTTTCAA -3'			
Serpina3n<br>Reverse: 5'-TTATCAGGAAAGGCCGATTG -3'			
**Critical commercial assays**			
Dulbecco's Modified Eagle Medium/Nutrient Mixture F-12	Gibco	Cat# 11330-032	N/A
Heat-inactivated fetal bovine serum	Gibco	Cat# 10099141	N/A
Penicillin and streptomycin	Gibco	Cat# 15140122	N/A

TRIzol reagent	Invitrogen,	CAT# 15596-026	N/A
PrimeScript™ RT Reagent Kit	TAKARA	CAT# RR047A	N/A
TB Green™ Premix Ex Taq™ II kit	TAKARA	CAT# RR820A	N/A
Adult Brain Dissociation Kit	Miltenyi Biotec	CAT# 130-107-677	N/A
Anti-ACSA-2 MicroBead Kit	Miltenyi Biotec	CAT#130-097-67	N/A
**Software and algorithms**			
GraphPad Prism 8	GraphPad Software	N/A	RRID:SCR_002798
ImageJ	https://imagej.net/	N/A	RRID:SCR_003070
